# A KO_2_‑Based Solid Superoxide Reservoir for Irreversible Capacity Compensation in Superoxide-Based Na–O_2_ Batteries

**DOI:** 10.1021/jacs.6c11101

**Published:** 2026-07-30

**Authors:** Cuiyi Deng, Yu Zhong, Lei Qin, Yiying Wu

**Affiliations:** † Institute for Advanced Study (IAS), 47890 Shenzhen University, Shenzhen 518060, PR China; ‡ Department of Chemistry and Biochemistry, 2647 The Ohio State University, 100 West 18th Avenue, Columbus, Ohio 43210, United States

## Abstract

Sodium–oxygen (Na–O_2_) batteries are plagued by persistent discharge–charge capacity imbalances stemming from parasitic reactions and the incompletely reversible oxygen redox chemistry induced by the reactive superoxide species. Herein, we propose the integration of commercial potassium superoxide (KO_2_) as a sacrificial solid reservoir to actively compensate for the rechargeable superoxide loss in sodium superoxide (NaO_2_)-based Na–O_2_ batteries. Mechanistic and spectroscopic investigations reveal that the preloaded KO_2_ serves a dual function: it acts as an extra superoxide source to directly replenish early stage capacity losses while simultaneously functioning as a promoter to mediate the formation of NaO_2_. Benefiting from this reservoir-mediated regulation, the KO_2_-configured Na–O_2_ cells exhibit unprecedented cyclability, delivering a near-unity Coulombic efficiency (98.5% on average) and sustaining a prolonged lifespan of over 600 cycles. This work establishes a robust paradigm for active capacity compensation, offering a highly scalable design principle to unlock the full potential of alkali metal–air battery systems.

## Graphical abstract

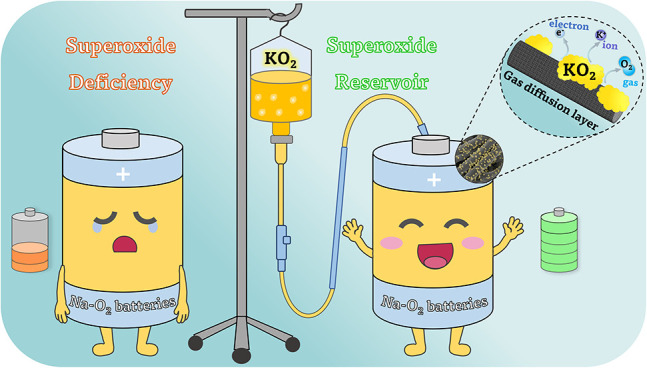

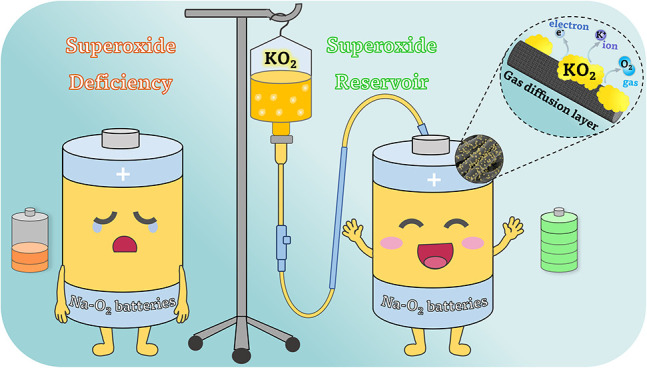

## 1 Introduction

Rechargeable sodium–oxygen (Na–O_2_) batteries represent a promising frontier for next-generation energy storage, potentially surpassing current lithium-ion battery technologies by virtue of their high theoretical specific energy and the elemental abundance of both sodium and oxygen.^1^ Compared with peroxide-based chemistries (e.g., in lithium–oxygen batteries), the superoxide chemistry that yields sodium superoxide (NaO_2_) as the primary discharge product (O_2_ + Na^+^ + e^–^ → NaO_2_, *E* ^⊖^ = 2.27 V vs Na^+^/Na) features a lower decomposition overpotential and faster reaction kinetics without the need for electrocatalysts.^1^ Nevertheless, the practical viability of superoxide-based Na–O_2_ batteries remains thwarted by the thermodynamic instability of NaO_2_. This reactive species frequently undergoes disproportionation to form sodium peroxide (Na_2_O_2_) or participates in nucleophilic attacks on electrolyte components. Such parasitic pathways lead to the accumulation of irreversible byproducts (e.g., Na_2_O_2_ and other sodium-containing oxygenated species), continuous consumption of electrolyte, and the gradual exhaustion of the active sodium (Na) inventory and the rechargeable superoxide portion.^2^ These chemical instabilities are macroscopically manifested as a limited Coulombic efficiency (CE), severe voltage polarization, and a rapidly decaying cycle life for practical Na–O_2_ batteries.

Extensive research efforts have sought to overcome these limitations through electrolyte engineering, microstructural design of the gas diffusion layer (GDL), and integration of cathode catalysts.^3–7^ While these strategies partially mitigate parasitic pathways or accelerate reaction kinetics, they often entail complex fabrication procedures and elevated costs. More fundamentally, these conventional preventative approaches offer no remedy for the cumulative loss of active Na inventory once irreversible byproducts form. Inspired by the cathode compensation concept, we propose a conceptually distinct strategy: introducing an internal solid reservoir to sustainably replenish decomposable superoxide species. Such a sacrificial superoxide reservoir can seamlessly compensate for the net capacity loss, thereby sustaining the charge–discharge capacity balance despite unavoidable parasitic reactions. Commercial potassium superoxide (KO_2_) emerges as an ideal candidate of the superoxide reservoir due to its ready availability, high superoxide density, and intrinsic stability.^8^ Notably, KO_2_ exhibits a decent solubility in common organic solvents. This characteristic facilitates the formation and decomposition of KO_2_ via a solution-mediated pathway, thereby minimizing the round-trip overpotentials (ca. 60 mV at moderate current densities) in potassium–oxygen batteries.^9^ In comparison, peroxide-based lithium–oxygen batteries suffer from limited reversibility with ultrahigh overpotentials exceeding 1.0 V, which severely hinders their practical application.^10^ While trace KO_2_ dissolution has been shown to favorably mediate discharge–charge kinetics in lithium–oxygen battery systems via a solution-phase mechanism,^11^ its potential to serve simultaneously as a bulk superoxide reservoir and a kinetic promoter in Na–O_2_ batteries has not been established. Revealing this behavior could unlock a scalable pathway toward the highly reversible Na–O_2_ chemistry.

Herein, we demonstrate a simple yet scalable strategy to circumvent the persistent irreversible capacity loss in superoxide-based Na–O_2_ batteries by utilizing commercial KO_2_ as a sacrificial solid superoxide reservoir. By preloading KO_2_ onto the GDL via a facile drop-casting method, we provide a stoichiometrically controllable and decomposable superoxide equivalent that replenishes the active superoxide source during the charging process. Mechanistic investigations reveal that this reservoir-mediated regulation also effectively suppresses byproduct accumulation and stabilizes the desirable NaO_2_ formation/decomposition pathway. Consequently, the Na–O_2_ battery achieves unprecedented reversibility (average CE = 98.5%) and sustains over 600 stable cycles. This approach offers a practical and catalyst-free route to sustain electrochemical reversibility, providing a robust paradigm for active capacity compensation in high-energy-density metal–air battery systems.

## 2 Results and Discussion

A central consideration in this work addresses the active Na inventory dilemma inherent to Na–O_2_ batteries. Because the pristine cathode lacks an active Na source and the electrolyte contains only a minor Na fraction (1%–5%) dedicated primarily to ionic conduction, the anode serves as the exclusive supplier of Na inventory. The conventional practice of employing a vast excess of Na metal often masks the underlying irreversibility of the cell and further reduces the achievable energy density from the battery aspect. Specifically, when the CE is significantly below 100%, each charge–discharge cycle inflicts a net deficit of reversible Na source (Scheme 1, upper panel). For instance, if a specific amount of Na is consumed during discharge but only a fraction is electrochemically recovered upon charging, the overall Na inventory undergoes continuous depletion. To mitigate this issue, our design integrates the commercial KO_2_ as an extra superoxide reservoir into the cathode GDL (Scheme 1, lower panel). Even upon the accumulation of irreversible Na source at the cathode, the replenishment from the decomposable superoxide sustains sufficient charge capacity, thereby preserving the reversible Na inventory at the anode.

**Scheme 1:**
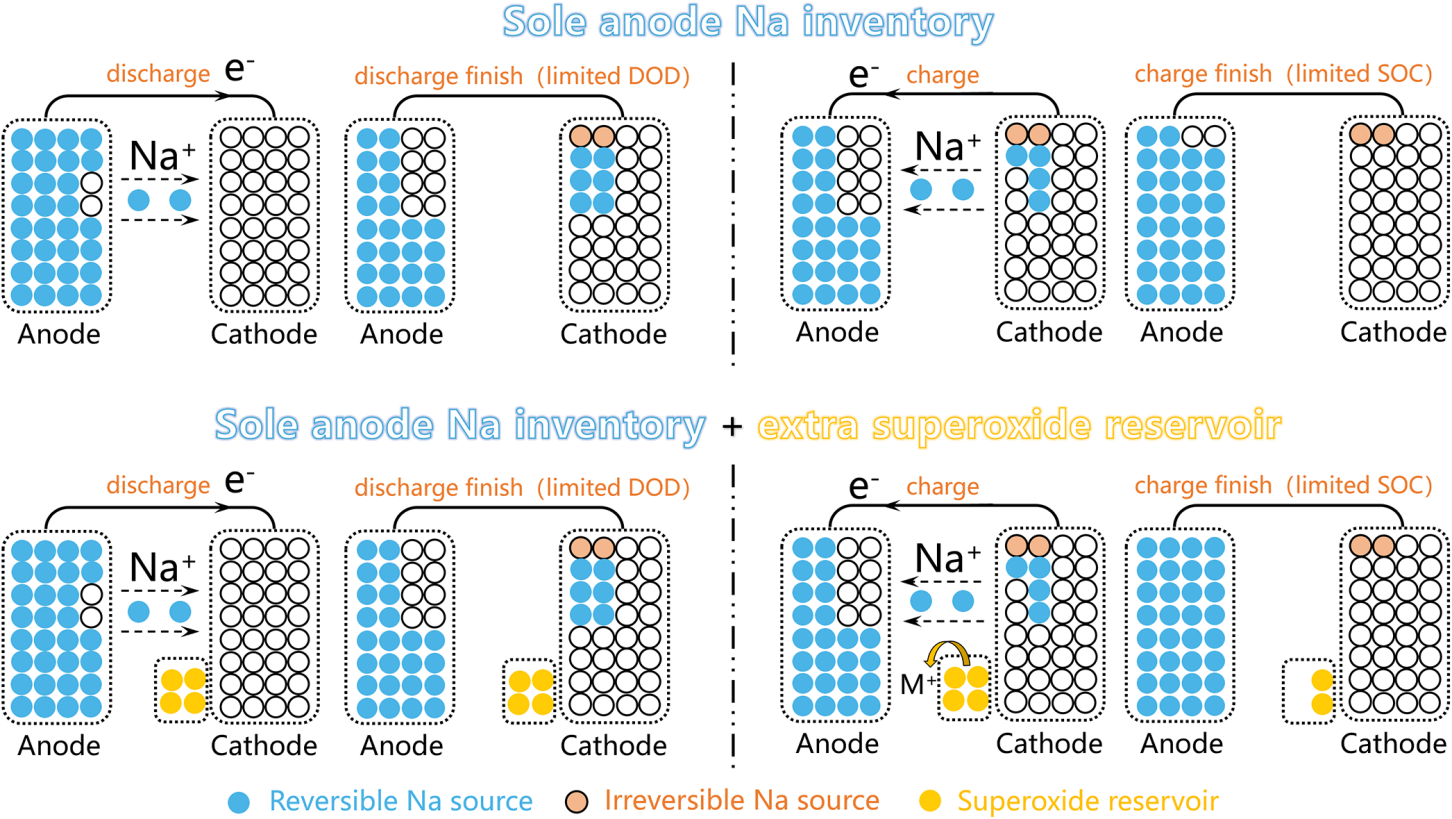
Schematic Comparison of the Active Na Inventory Evolution in Na–O_2_ Batteries with and without an Extra Superoxide Reservoir^a^

a Reversible Na source predominantly comprises metallic Na at the anode and NaO_2_ at the cathode, whereas the irreversible Na source represents nondecomposable and Na-containing oxygenated byproducts (e.g., acetates, formates, and peroxides). The superoxide reservoir consists of electrochemically decomposable alkali metal superoxide (e.g., NaO_2_ and KO_2_). DOD: depth of discharge. SOC: state of charge.

The compatibility of the KO_2_-preloaded GDL with the Na–O_2_ battery chemistry was initially evaluated by characterizing the discharge products (Figure 1). For the baseline cell utilizing the pristine P50 GDL and 05NG electrolyte, the XRD pattern exclusively identifies crystalline NaO_2_ as the predominant discharge product, devoid of any discernible peroxide or hydroxide impurities (blue trace, Figure 1d). Cubic NaO_2_ species are randomly distributed within the porous carbon network of the P50 GDL (Figure 1c, Figures S1–S2). Similarly, the P50@KO_2_ GDL exhibits micrometer-scale cubic NaO_2_ products upon discharge (Figure 1a–b). EDS elemental mapping reveals the spatial colocalization of Na and O signals, which are observed both on the surface of granular KO_2_ aggregates and within the intrinsic voids of the carbon matrix (Figure S3). The coexistence of preloaded KO_2_ and newly nucleated NaO_2_ is further substantiated by the XRD pattern (cyan trace, Figure 1d). These results confirm that the incorporation of KO_2_ does not perturb the desirable one-electron superoxide chemistry during the discharge process of the constructed Na–O_2_ battery.

**Figure 1:**
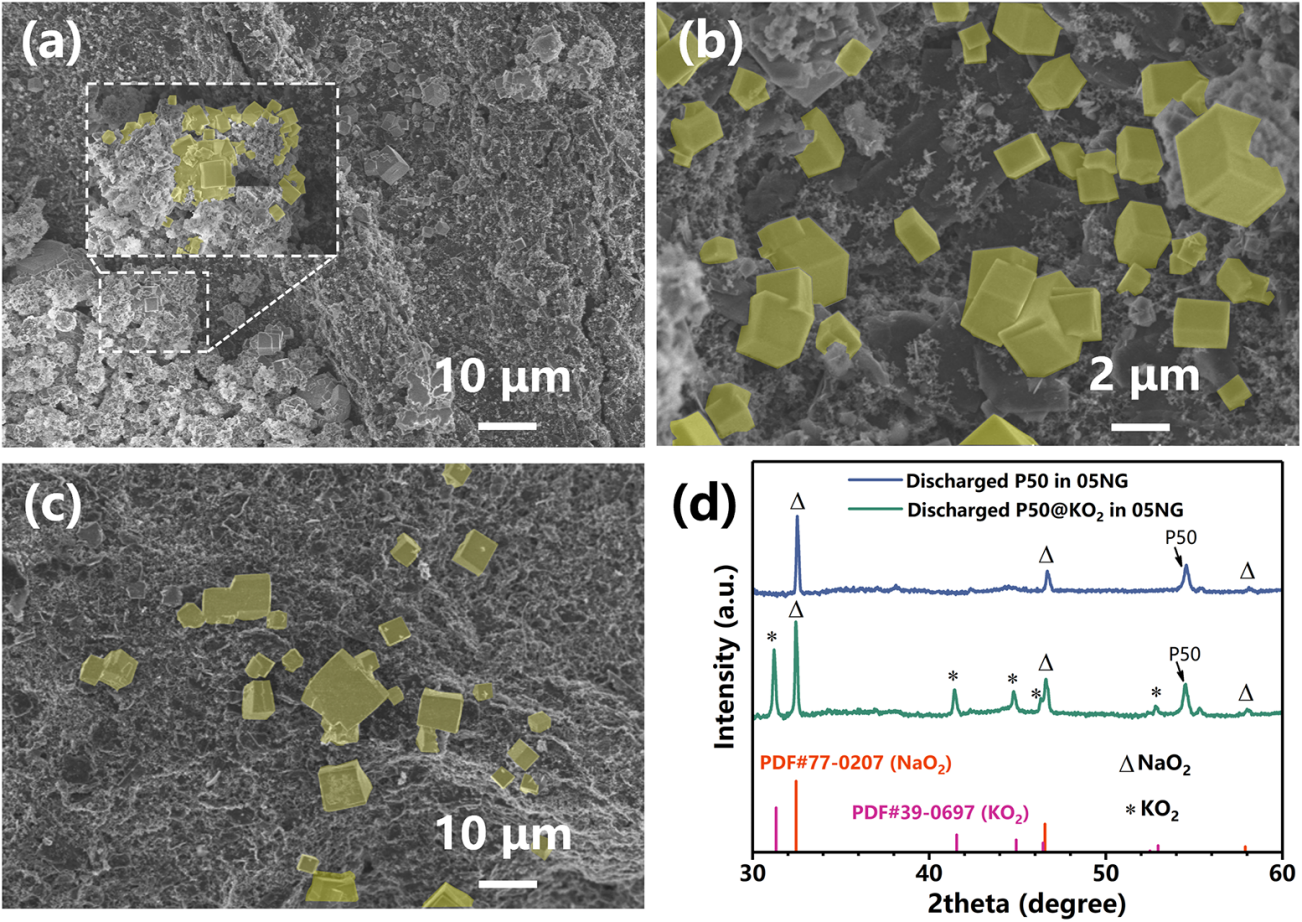
Morphological and structural characterization of discharge products. (a–c) Representative SEM micrographs of the cathode GDLs discharged to a capacity of 1.0 mAh: (a, b) P50@KO_2_ GDL at different magnifications and (c) P50 GDL. (d) XRD diffraction patterns of the products harvested from cells using the 0.5 M NaOTf/G2 (05NG) electrolyte, confirming the crystalline phases within the porous GDL matrix.

To elucidate the superoxide decomposition sequence, the equilibrium potentials of NaO_2_/O_2_ and KO_2_/O_2_ redox couples were determined using a three-electrode configuration with the ferrocenium/ferrocene (Fc^+^/Fc) as an internal reference (Figure S4). The NaO_2_/O_2_ and KO_2_/O_2_ couples exhibit equilibrium potentials of −1.01 V and −1.15 V vs Fc^+^/Fc, respectively. These values translate to −0.61 V and −0.75 V vs SHE, assuming a standard potential offset of 0.40 V. The more negative redox potential of the KO_2_/O_2_ couple indicates a stronger driving force for electron loss compared to NaO_2_/O_2_, suggesting that KO_2_ should undergo preferential decomposition when both species coexist. This thermodynamic prediction is further corroborated by time-resolved *in situ* XRD analysis (Figure 2a). Monitoring the characteristic reflections of KO_2_ (2θ = 31.1°) and NaO_2_ (2θ = 32.3°) reveals distinct kinetic behaviors during the incipient stages of charging. Specifically, the KO_2_ peak intensity decreases by 14.5% within the first 100 min, whereas the NaO_2_ intensity remains at 98.7% of its initial value (Figure S5). These results establish KO_2_ as a kinetically privileged and compensatory superoxide source that facilitates the early stage charging process.

**Figure 2:**
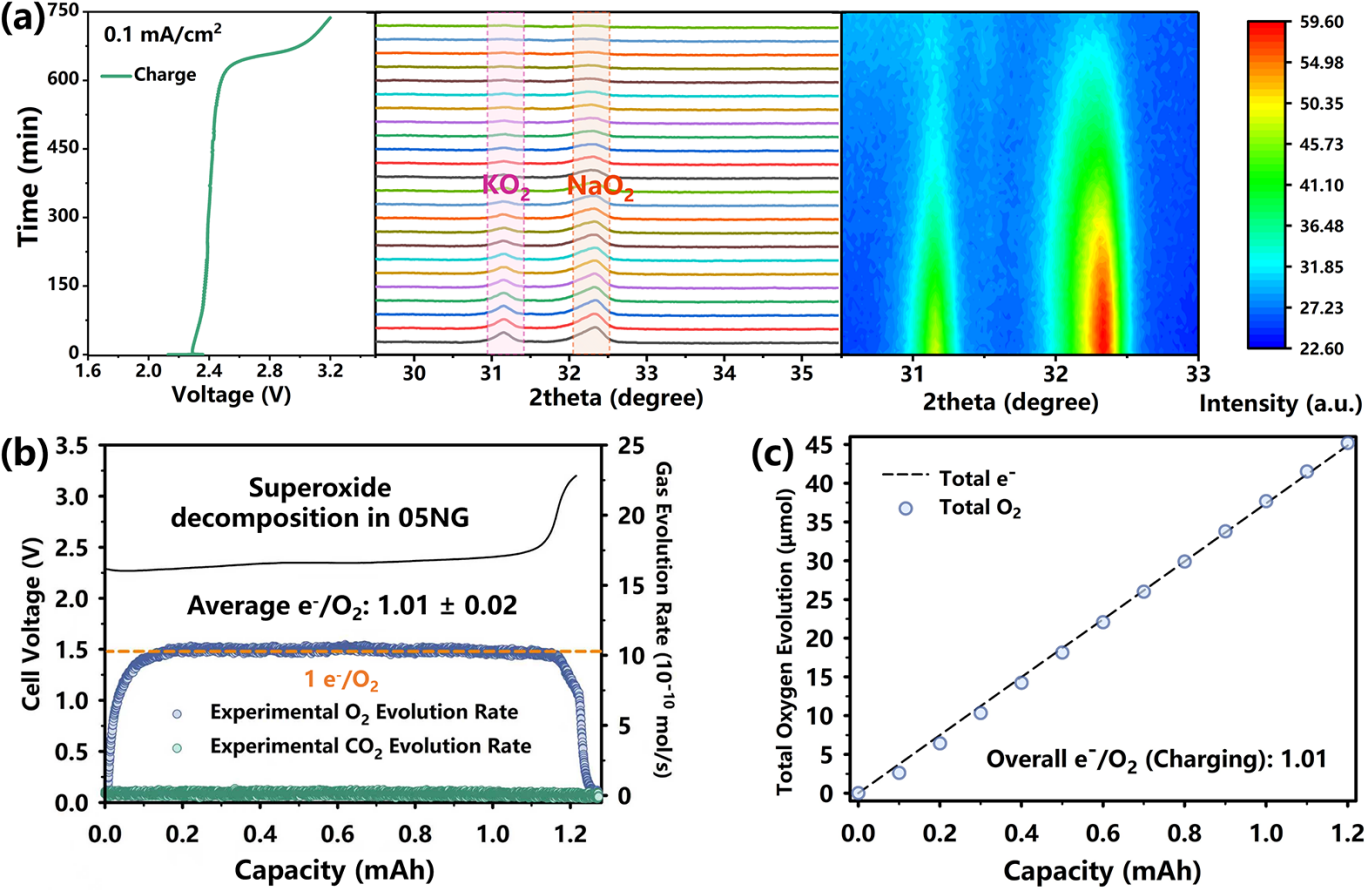
Mechanistic investigation of the superoxide evolution process. (a) *In situ* XRD and (b, c) *in situ* DEMS characterization of the P50@KO_2_/NaO_2_ cathode during the charging process (0.1 mA/cm^2^ to 3.2 V): (a) The charge profile (left), stacked plots (middle), and 2D color-filled contour plot (right) of XRD patterns. (b) Time-resolved evolution rates of individual gaseous species during the DEMS measurement. (c) Cumulative oxygen evolution as a function of different SOCs. The superoxide-enriched P50@KO_2_/NaO_2_ electrode was prepared by discharging the P50@KO_2_ GDL (preloaded with 80 μL of KO_2_/G1 suspension) to a capacity of 1.0 mAh in a Na–O_2_ cell using 05NG electrolyte.

To investigate potential parasitic reactions and elucidate the electrochemical charging mechanism, gas evolution rates (O_2_ and CO_2_) were monitored via *in situ* DEMS (Figure 2b). The measured O_2_ evolution rate closely aligns with the theoretical value for a one-electron process (10.38 × 10^–10^ mol/s) throughout the majority of the charging process. The value is calculated via *I*/*F*, where the parameter *I* is the applied current and *F* is the Faraday constant. Subsequently, the rate declines significantly to 2.60 × 10^–10^ mol/s as the charging reaches its conclusion. Notably, no discernible CO_2_ is detected, precluding the decomposition of carbonate-based byproducts. Furthermore, these results validate the electrochemical stability of both electrolyte and carbon-based GDL below the upper voltage threshold of 3.2 V. By integrating the cumulative O_2_ evolution relative to the SOC, an overall e^–^/O_2_ ratio of 1.01 is obtained (Figure 2c). This stoichiometric correlation confirms that the charging capacity is exclusively governed by the single-electron oxidation of superoxide species, free from interference by parasitic side reactions.

Notably, the Na–O_2_ battery utilizing the P50@KO_2_ GDL exhibits a first-cycle charge capacity of 1.75 mAh, significantly surpassing that of the pristine P50 counterpart (0.64 mAh, Figure 3a), despite both being initially discharged to 1.0 mAh (Figure S6). This pronounced disparity is ascribed to the additional capacity contribution from the oxidative decomposition of the preloaded KO_2_. To further elucidate the influence of K^+^ ions released during this process, a nominal K^+^ concentration of 0.18 M is determined based on the KO_2_ loading (see details in the note of Figure S6). Subsequently, a control electrolyte containing 0.5 M NaOTf + 0.18 M KOTf in G2 (denoted as 05NG-K) was evaluated with a blank P50 GDL, which displays a discharge profile nearly identical to that of the 05NG baseline (Figure 3a). ICP-OES analysis was performed to quantify the elemental distribution within the electrolytes at specific states (Figure 3b). The elemental Na/K mass ratio in the 05NG & P50@KO_2_ cell after the first charge is 1.64, substantially lower than that of 2.52 observed in the discharged 05NG-K & P50 cell. This contrast confirms the thorough decomposition of the preloaded KO_2_ and the resultant K^+^ release into the electrolyte.

**Figure 3:**
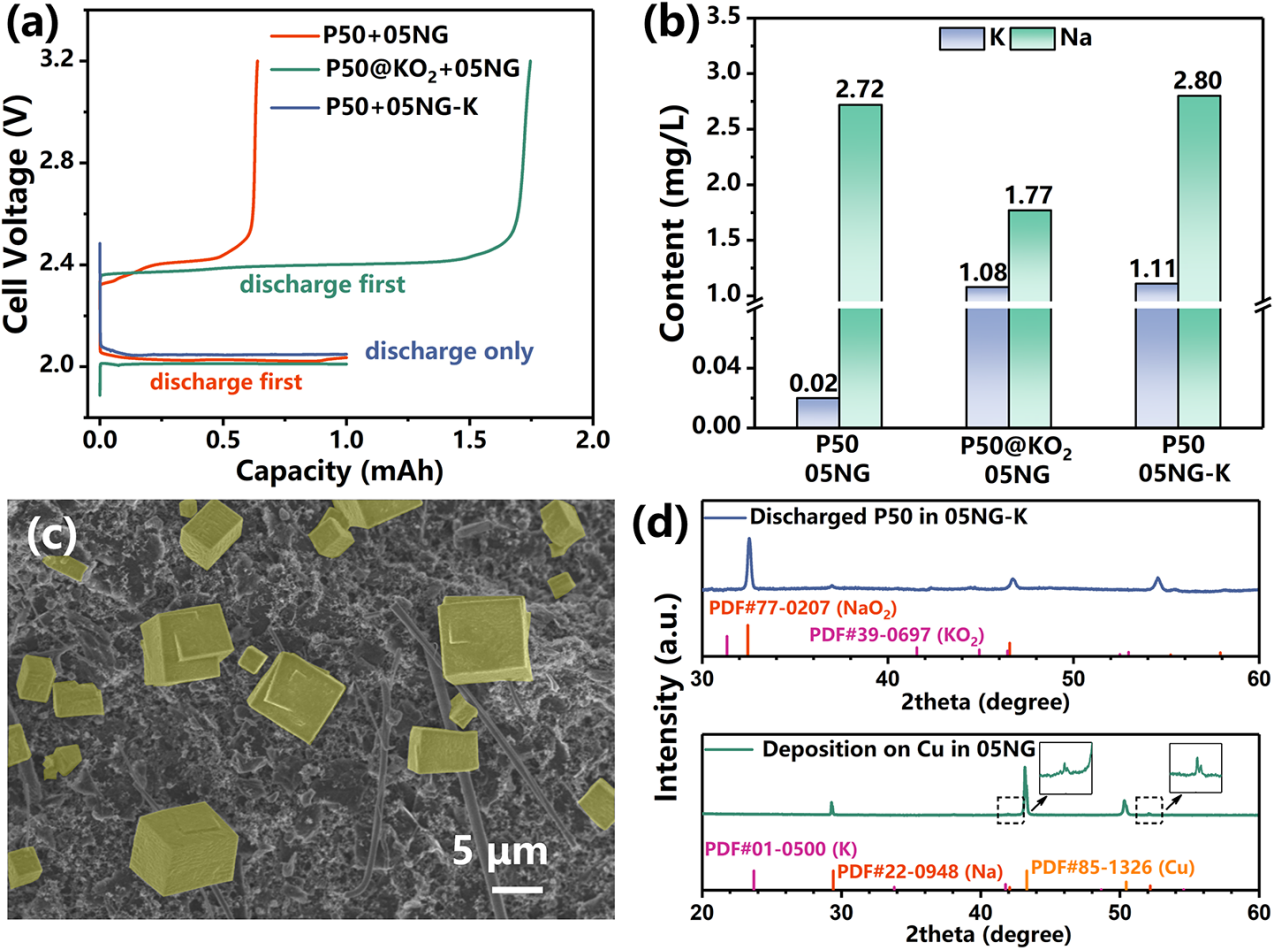
Investigation of K-species migration and its impact on the electrode electrochemistry of Na–O_2_ batteries. (a) Representative 1st galvanostatic discharge–charge profiles of Na–O_2_ batteries utilizing P50 GDL (red trace) and P50@KO_2_ GDL (cyan trace) in 05NG electrolyte. The discharge profile of a P50 GDL-based cell in a K^+^-containing electrolyte of 05NG + 0.18 M KOTf (05NG-K; blue trace) is included for comparison. (b) Elemental analysis (ICP-OES) of Na and K species accumulated on the cycled separators from the respective cells in (a). (c) SEM image and (d) XRD pattern of the discharged P50 GDL using 05NG-K electrolyte (upper panel). The lower panel in (d) shows the XRD reflection of the metal deposited on a bare Cu substrate in 05NG electrolyte, where the superoxide-enriched electrode of P50@KO_2_/NaO_2_ serves as the counter electrode.

To investigate whether the K^+^ ions released from KO_2_ decomposition competitively associate with superoxide ion intermediates to form KO_2_ precipitates, the cathode from a 05NG-K & P50-based cell was systematically characterized after the first discharge of a Na–O_2_ battery. SEM micrographs reveal that the discharge products consist exclusively of cubic-shaped solids (Figure 3c). Corresponding EDS elemental maps confirm the spatial colocalization of Na and O signals, with no detectable K-related species within these cubic structures (Figure S7). This is further corroborated by XRD analysis (Figure 3d, upper panel), where the reflections correspond solely to the crystalline NaO_2_ phase, devoid of any KO_2_ signatures. These findings suggest that despite the presence of K^+^, NaO_2_ remains the preferred discharge product. Indeed, trace concentrations of K^+^ are proposed to act as soluble superoxide promoters that stabilize the NaO_2_ product, rather than participating as a bulk component of the solid discharge species, consistent with a prior report.^12^

The influence of K^+^ ions, released from the KO_2_ reservoir, on the Na anode behavior was systematically evaluated. A discharged cathode (DOD of 1.0 mAh) was harvested from a P50@KO_2_-based cell to acquire the superoxide-enriched cathode of P50@KO_2_/NaO_2_, which was then reassembled into an asymmetric cell with bare Cu foil anode substrate and 05NG electrolyte to probe metal cation deposition. XRD analysis of the plated species exclusively identifies metallic Na, with no detectable signal from K metal (Figure 3d, lower panel). This observation aligns with thermodynamic predictions, as the higher reduction potential of Na^+^ over K^+^ ensures the predominance of Na plating (Figure S8). Consequently, K^+^ ions persist in the electrolyte as spectator species, providing a reasonable explanation for the elevated elemental K content measured via ICP-OES after charging (Figure 3b). Notably, alternating current voltammetry on Na–Cu half-cells reveals that trace K^+^ in the electrolyte could modulate the double-layer capacitance and induce a negative shift in the potential of zero charge, signifying the specific adsorption of K^+^ on the anode substrate (Figure S9). While this adsorption does not perturb SEI formation as revealed by linear sweep voltammetry, it likely promotes more uniform Na deposition via the electrostatic shielding mechanism. Collectively, these results demonstrate that K^+^ released from the sacrificial decomposition of KO_2_ remains chemically stable in the electrolyte, ensuring minimal interference with the Na stripping/plating kinetics and the NaO_2_ redox chemistry. Furthermore, the presence of trace K^+^ is conducive to enhancing electrode–electrolyte compatibility through the synergistic suppression of Na dendrite growth and superoxide-mediated degradation.

Long-term cyclic stability of the Na–O_2_ batteries was evaluated using a capacity-curtailed strategy (DOD of 0.2 mAh/cm^2^ at 0.1 mA/cm^2^). The control cell utilizing the 05NG electrolyte and a P50 GDL exhibits a gradual activation process with CEs increasing from 51.8% (first cycle) to 84.2% by the 15th cycle (Figure 4a and Figure 4c). Subsequently, the CE values stabilize at approximately 87–90% (50th CE = 87.1%, 100th CE = 89.7%, 300th CE = 89.2%, 420th CE = 88.8%) before the cell suffers from sudden failure after 420 cycles, implying the limited electrode–electrolyte compatibility. In stark contrast, the incorporation of the P50@KO_2_ GDL endows the Na–O_2_ battery with a consistent CE of 100% during the initial 73 cycles (Figure 4b–c). This near-unity efficiency is ascribed to the capacity compensation provided by the sacrificial decomposition of the preloaded KO_2_, as corroborated by our spectroscopic and electrochemical analyses. The relationship between KO_2_ loading and the compensation effect is further elucidated by systematic capacity analysis (Figure S10). This optimization confirms an optimal loading of 150 μL to ensure maximum reservoir utilization efficiency. Nitrogen adsorption–desorption measurement analysis confirms that the KO_2_-preloaded P50 GDL retains the original microstructure framework of the P50 GDL, despite a minor reduction in the specific surface area and pore volume (Figure S11). Furthermore, the P50@KO_2_-based battery delivers a prolonged lifespan exceeding 600 cycles (over 2400 h stable operation) with an exceptional average CE of 98.5%. Notably, the cell also maintains a high average energy efficiency (EE) of 87.3% throughout its lifespan (Figure 4d), implying accelerated reaction kinetics at both electrode–electrolyte interfaces. The slightly larger voltage gaps observed during the initial 30 cycles are likely associated with interfacial stabilization processes. Full discharge–charge tests at the higher current density (0.4 mA/cm^2^) further validate the benefits of KO_2_. The baseline cell fails within 3 cycles due to recurrent microshort circuits, while the P50@KO_2_ system exhibits significantly ameliorated capacity retention and stable voltage profiles (Figure S12). Comparative rate performance tests demonstrate that P50@KO_2_ electrodes exhibit superior capacity retention and lower polarization than pristine P50 at various current densities (Figure S13, Table S1). These results indicate that the KO_2_ reservoir improves electrochemical performance primarily through compensation effects and enhanced electrode–electrolyte compatibility.

**Figure 4:**
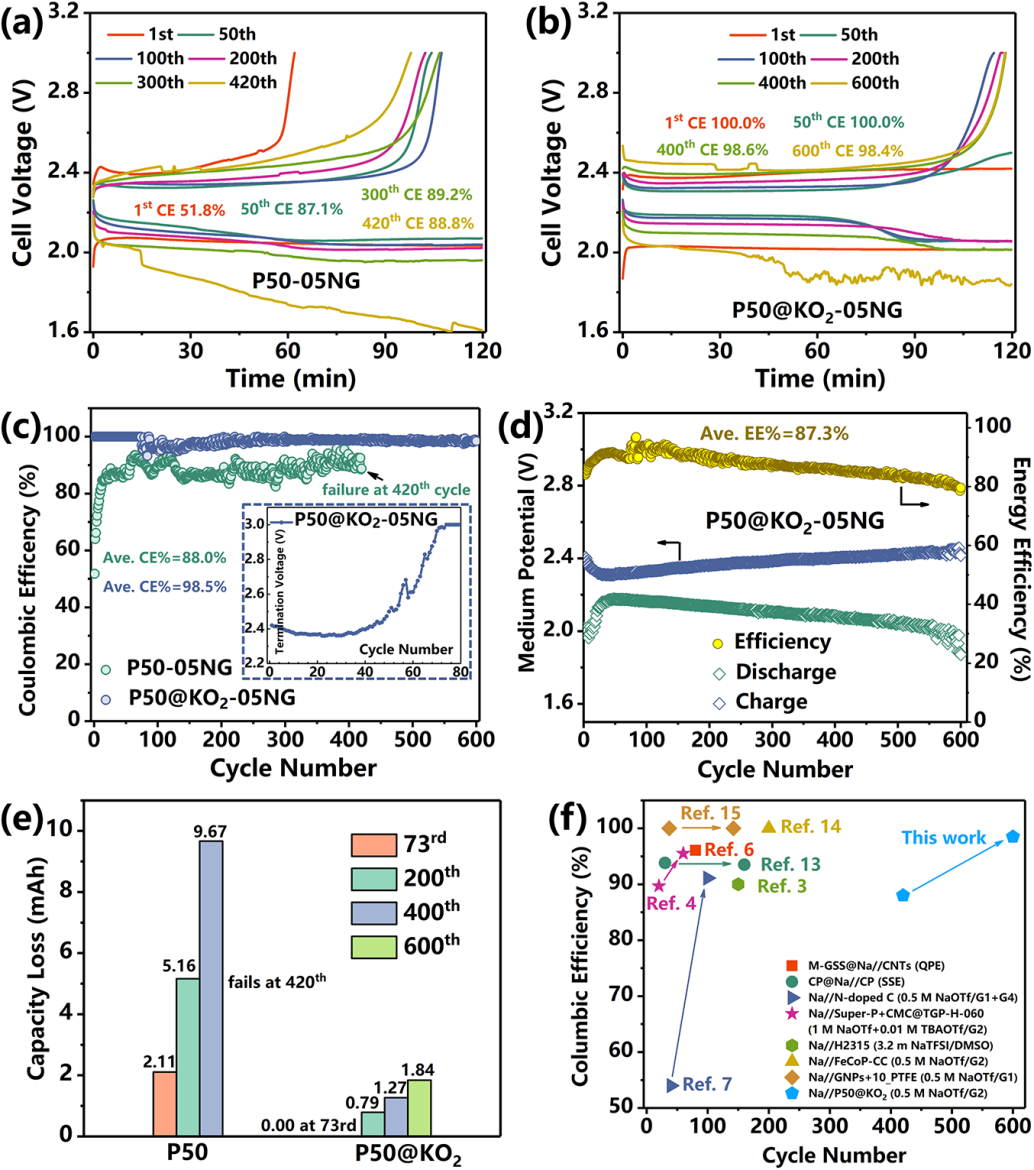
Enhancement of Na–O_2_ battery performance via KO_2_ preloading. (a, b) Typical galvanostatic voltage profiles of Na–O_2_ batteries utilizing (a) a P50 GDL and (b) a P50@KO_2_ GDL (preloaded with 150 μL of KO_2_/G1 suspension) in 05NG electrolyte. (c) Long-term CE comparison over 600 cycles. The inset shows the corresponding charge cutoff potentials for the P50@KO_2_ system. (d) Evolution of medium discharge/charge potentials and the resulting energy efficiencies of the P50@KO_2_-based battery. (e) Comparative capacity loss analysis of Na–O_2_ batteries. (f) Performance benchmark (CE vs cycle number) of the current work compared with state-of-the-art Na–O_2_ batteries reported recently.^3,4,6,7,13–15^

The cumulative capacity losses of Na–O_2_ batteries utilizing P50 and P50@KO_2_ GDLs were further quantified based on the CE data from Figure 4c and compared in Figure 4e. At the 73rd, 200th, and 400th cycles, the accumulated capacity losses for the P50 baseline are 2.11, 5.16, and 9.67 mAh, respectively. In stark contrast, these values are significantly reduced to 0, 0.79, and 1.27 mAh for the P50@KO_2_ system. Notably, the zero capacity loss observed during the initial 73 cycles confirms that the preloaded KO_2_ effectively compensates for early stage irreversible losses, enabling a near-unity CE of 100%. Furthermore, the P50@KO_2_-based battery maintains stable operation for over 600 cycles with a total capacity loss of only 1.84 mAh. These results demonstrate that the introduction of KO_2_ effectively mitigates irreversible capacity decay and attenuates active Na source depletion, which can be attributed to the crucial role of KO_2_ as the rechargeable superoxide reservoir and the desirable NaO_2_ promoter discussed above. As illustrated in Figure 4f, this work achieves a superior battery lifespan (>600 cycles) and exceptional reversibility (average CE = 98.5%) compared to state-of-the-art Na–O_2_ batteries reported recently (see Table S2 for details).^3,4,6,7,13–15^

To assess the practical viability of our design, we calculated the energy densities of the Na–O_2_ batteries based on the total mass of the active electrode components, including the Na metal anode, the P50 GDL, the NaO_2_ discharge products, and the preloaded KO_2_ (where applicable) (Figure S14, Table S3). The P50@KO_2_ system achieves a specific energy of 151.27 Wh/kg, outperforming the P50 benchmark (119.34 Wh/kg) despite the mass contribution of the KO_2_ reservoir (5.8 wt %). Additionally, the cumulative areal capacity of the P50@KO_2_ battery reaches 126.32 mAh/cm^2^ over 600 cycles, higher than the 88.42 mAh/cm^2^ obtained from the P50 benchmark (420 cycles). Besides, the Na–O_2_ battery with the P50@KO_2_ GDL demonstrates a superior cost-performance ratio, reflected in a reduced price per unit of energy output (Table S4). These improvements highlight the effectiveness of the KO_2_ reservoir in extending cycle life and increasing overall specific energy. Analysis of the failure mechanism reveals that anode deactivation due to oxygen crossover is the primary limiting factor. This is validated by a refreshing experiment (Figure S15), where cells recover stable voltage profiles upon replacing the cycled Na anode. Therefore, it is reasonable to anticipate that the proposed superoxide reservoir strategy would exert even greater potential once the challenge of stabilizing the Na metal anode is addressed, further elevating the practical viability of Na–O_2_ battery systems.

The efficacy of commercial Na_2_O_2_ as a sodium-based sacrificial additive was also investigated. Note that the commercial unavailability of NaO_2_ stems from its thermodynamic instability at room temperature. Although Na_2_O_2_ serves as a readily accessible alternative, it fails to act as an efficient compensation agent. The cell utilizing the P50@Na_2_O_2_ GDL exhibits significantly diminished performance, characterized by a truncated lifespan (41 cycles) and inferior reversibility (average CE = 73.2%) compared to the bare P50 benchmark (Figure S16). This contrast highlights that the capacity compensation is exclusive to superoxide chemistry rather than a generic effect of introducing oxygen-containing sacrificial solids. Specifically, insulating peroxide solids like Na_2_O_2_ involve peroxide chemistry and are subject to fundamentally different kinetic constraints, where severe electronic transport bottlenecks and a dominant surface-mediated pathway restrict efficient oxidative decomposition. In contrast, KO_2_ functions as an effective one-electron reservoir, providing a dedicated superoxide redox equivalent to compensate for capacity loss. The KO_2_ additive provides significantly enhanced decomposition kinetics via a unique solution-mediated pathway. As established in our recent study,^16^ this mechanism allows KO_2_ to remain electrochemically active even in the absence of additional conductive carbon, underscoring its superiority as a solid-state superoxide reservoir. Furthermore, the liberated K^+^ ions promote desirable NaO_2_ phase evolution and stabilize Na deposition. Consequently, selecting KO_2_ as a solid-state superoxide reservoir is well-justified for enhancing superoxide-based Na–O_2_ battery performance.

As a final note, we compare the KO_2_ reservoir strategy with the established prelithiation additive concept in rechargeable batteries (Table S5). These additives generally include lithium metals, lithium metal oxides,^17^ lithium metalloids, and lithium nonmetallic compounds (e.g., Li_2_O^18,19^ ). This comparison effectively positions the present work as an extension of sacrificial inventory compensation from standard lithium inventory management in lithium-ion batteries to superoxide inventory management in Na–O_2_ batteries. As advanced energy storage evolves, balancing safety, performance, and technological integration becomes paramount. Undoubtedly, the related study would pave the way for the next generation of high-performance lithium-ion batteries through prelithiation methods or post lithium-ion batteries (e.g., Na–O_2_ batteries) via the superoxide inventory replenishment.

## 3 Conclusions

In summary, we have demonstrated a conceptually distinct and scalable strategy to mitigate the persistent irreversible capacity loss in Na–O_2_ batteries by integrating commercially available KO_2_ as a sacrificial solid superoxide reservoir. Our findings reveal that KO_2_ exerts a synergistic effect: it functions as a stoichiometric superoxide source to replenish active inventory, and liberates K^+^ ions that stabilize the Na anode interfacial kinetics and act as a promoter to facilitate the formation of NaO_2_. Benefiting from this reservoir-mediated compensation, the Na–O_2_ system exhibits unprecedented reversibility and longevity, characterized by an average CE of 98.5% over 600 stable cycles. By bypassing the complexities of catalyst and electrode engineering, this work underscores the potential of chemical compensation in sustaining reactive oxygen redox chemistries. The concept of using a solid superoxide reservoir provides an alternative for compensating the oxygen-related capacity loss, offering a promising and extendable design principle for rechargeable alkali metal–oxygen batteries.

## Supplementary Material



## Notes

Experimental details for materials, battery assembly, and characterization methods; additional SEM images of electrode morphologies; XRD peak intensity evolution; N_2_ physisorption isotherms and BJH pore size distributions; electrochemical performance data (CV, ACV, LSV, galvanostatic tests); and supplementary tables listing cell component specifications, performance metrics, and compensation strategy comparisons (PDF)
