# Supplementary material for: A KO2‑Based Solid Superoxide Reservoir for Irreversible Capacity Compensation in Superoxide-Based Na–O2 Batteries
Source: J Am Chem Soc. 2026 Jul 30;148(31):33735–42. doi: 10.1021/jacs.6c11101 (PMC13474932; doi:10.1021/jacs.6c11101)
Supplement: Supplementary file 1 [file ja6c11101_si_001.pdf]

Supporting Information

for

A KO_2-Based Solid Superoxide Reservoir for Irreversible Capacity Compensation in Superoxide-Based Na–O_2 Batteries

Cuiyi Deng‡, Yu Zhong‡, Lei Qin, Yiying Wu**

C. Deng‡, Y. Zhong‡, L. Qin*

Institute for Advanced Study (IAS), Shenzhen University, Shenzhen 518060, PR China

Y. Wu*

Department of Chemistry and Biochemistry, The Ohio State University, 100 West 18th Avenue, Columbus, Ohio 43210, United States

‡These authors contributed equally to this work.

*Corresponding authors

E-mail: lei.qin@szu.edu.cn

E-mail: wu@chemistry.ohio-state.edu

1. Experimental section

1.1 Materials

Battery-grade sodium trifluoromethanesulfonate (NaOTf, Dodo) and potassium trifluoromethanesulfonate (KOTf, Dodo) were dried at 80°C under vacuum for 24 hours before use. 1,2-dimethoxyethane (DME, labeled as G1, Dodo) and diethylene glycol dimethyl ether (DEGDME, labeled as G2, Dodo) were dehydrated over activated 4 Å molecular sieves for one week, ensuring a residual water content below 10 ppm (measured by Karl Fischer titration). Metallic sodium (Na, Aladdin, ≥99.7%) and potassium (K, Aladdin, ≥99%) were mechanically cleaned to remove surface mineral oil and oxides. The purified metals were then processed into uniform discs of fixed thickness and diameter using a manual rolling press and a precision punch inside an argon-filled glovebox. Potassium superoxide (KO_2, Sigma-Aldrich), sodium peroxide (Na_2O_2, Alfa Aesar), and carbon paper gas diffusion layer (GDL) electrodes (AvCarb P50, Fuel Cell Store) were used as received.

1.2 Battery assembly and electrochemical characterization

The electrolyte solutions were prepared by weighing the requisite amounts of NaOTf or KOTf salt using a precise balance, followed by the addition of a measured volume of G2 solvent. The mixtures were vigorously stirred until completely dissolved to yield clear solutions within an argon-filled glovebox. The resulting formulations of 0.5 mol/L (M) NaOTf/G2 and 0.18 M KOTf/G2 are denoted as 05NG and 018KG, respectively. The KO_2-preloaded GDL (P50@KO_2) was fabricated via a suspension drop-casting technique, as previously reported.[1] Briefly, a 20 mg/mL KO_2/G1 suspension was prepared and subsequently drop-cast onto both sides of the P50 substrate (**Figure S1**). The Na_2O_2-preloaded counterpart (P50@Na_2O_2) was synthesized using an identical protocol, substituting the precursor with a 20 mg/mL Na_2O_2/G1 suspension.

Na–O_2 batteries were assembled using custom-engineered Swagelok-type cells. The anode consists of metallic Na pressed onto bare Cu foil (Canrd), while the cathode is composed of

commercial P50 or P50@KO_2 carbon paper GDLs. The separator is a composite of one layer of glass fiber filter (Whatman, GF/D) and a tri-layer Celgard 2325 membrane. Approximately 240 μL of electrolyte was used to ensure complete wetting of both the separator and the electrodes. All electrochemical evaluations were performed via galvanostatic measurements on a NEWARE battery test system (WHW-200L2-0C-220V-9U-PC, Shenzhen, China). The cells were maintained under isothermal conditions at 25℃ within a thermostatic oven. For cyclic stability tests, a capacity-limited protocol was employed at a current density of 0.1 mA/cm^2 with a fixed depth of discharge (DOD) of 0.2 mAh/cm^2 within a voltage window of 1.6–3.0 V. For long-term cycling tests, the P50@KO_2 GDL was prepared with a 150 μL KO_2/G1 suspension loading. For estimating the relationship between KO_2/G1 suspension loading and the resulting compensation effect, different volumes (50/80/100/150/200/250/300 μL) of KO_2/G1 were added to P50 GDLs and the assembled cells were charged to 3.0 V at 0.1 mA/cm^2. Full discharge–charge profiles were recorded at various current densities (0.2/0.3/0.4/0.5 mA/cm^2) between 1.6 and 3.0 V.

Cyclic voltammetry (CV) measurements were performed using an electrochemical workstation (CHI760E, Chenhua). A three-electrode configuration was employed to calibrate the redox potentials of target couples against the ferrocenium/ferrocene (Fc^+/Fc) internal standard in specific electrolyte recipes. The measurements were conducted in a five-neck electrolytic cell using a glassy carbon working electrode and a platinum wire counter electrode. The electrolytes comprise 0.18 M KOTf or 0.5 M NaOTf in G2 with the addition of 0.1 M ferrocene. To measure superoxide/oxygen (MO_2/O_2) potentials, as-prepared P50@MO_2 (M = Na, K) electrodes serve as the reference. The P50@NaO_2 electrode was obtained by discharging a Na–O_2 cell to a DOD of 1 mAh/cm^2, while the P50@KO_2 electrode was fabricated via the aforementioned drop-casting method. The electrolyte was purged with high-purity oxygen for 5 minutes before CV measurements, and scan ranges were set to 0.5–2.0 V (K-system) and 0.5–1.6 V (Na-system), respectively. For metal-ion/metal (M^+/M) potential calibrations, a metallic Na or K disc pressed onto Cu foil (Cu@M) was utilized as the reference electrode under an argon atmosphere, and CV measurements were then performed with a potential range of 2.5~4.0 V.

Linear sweep voltammetry (LSV) curves of Na–Cu half-cells were recorded from 0~2.0 V at a cathodic scan rate of 5 mV s^{-1}. Alternating current voltammetry (ACV) measurements of the Na–Cu half-cells were conducted with a perturbation amplitude of 25 mV and a frequency of 6 Hz. The potential was scanned cathodically from 2.0 to 1.5 V (vs. Na/Na^+). Phase angles of 0° and 90° were selected for analysis. The capacitance was determined according to the following relationship[2]:

$$Z = Z_{Re} + iZ_{Im} = R + (iCw)^{-1}; C = -(wZ_{Im})^{-1}$$

1.3 Characterization

All cells were disassembled inside an argon-filled glovebox (H_2O, O_2< 0.1 ppm). The cycled GDL electrodes were thoroughly rinsed with G1 to eliminate residual salts before further characterization. Morphological evaluation of the discharge products was performed using a high-resolution scanning electron microscope (SEM, Hitachi SU8010), while elemental distributions were determined via energy-dispersive X-ray spectroscopy (EDS). To prevent air exposure, all air-sensitive samples were stored in an airtight sample holder during transport to the SEM chamber. The elemental contents (specifically Na and K) within the electrolyte were quantified via an inductively coupled plasma optical emission spectrometer (ICP-OES, Arcos II MV). For ICP analysis, the cycled separators were retrieved and immersed in 3 mL G2 to ensure complete extraction of the electrolyte species. The resulting suspension was subsequently filtered through a membrane filter to yield a clear filtrate for measurements.

XRD patterns of the GDL electrodes were recorded on a Rigaku SmartLab diffractometer using Cu Kα radiation at a scan rate of 10°/min. To prevent ambient air exposure, the electrodes were hermetically sealed with a thin layer of self-adhesive polyimide tape before measurement. *In situ* XRD measurements were performed using a Bruker D8 ADVANCE diffractometer over a 2θ range of 29° to 39° at a scan rate of 1°/min. Simultaneously, the cell was subjected to a constant charging current density of 0.1 mA/cm^2 until a cutoff voltage of 3.2 V was reached. N_2 adsorption–desorption isotherms were recorded on a BELSORP-max instrument. The specific surface areas

were determined by the Brunauer–Emmett–Teller (BET) method, and the pore size distributions were obtained from the Barrett–Joyner–Halenda (BJH) analysis.

To monitor the gas evolution dynamics, a Na–O_2 battery was first galvanostatically discharged to a fixed capacity of 1.0 mAh to deposit NaO_2 onto the P50@KO_2 cathode, forming a P50@KO_2/NaO_2 composite GDL. The cell was assembled in an argon-filled glovebox using a custom-engineered Swagelok-type cell specifically designed for differential electrochemical mass spectrometry (DEMS) integration. A stainless-steel grid was incorporated to ensure efficient gas diffusion. Before measurement, the gas circuits were purged with high-purity argon at 7.0 mL/min to achieve a clean baseline. During the galvanostatic charge (0.1 mA/cm^2, up to 3.2 V), the carrier gas flow was maintained at 1.0 mL/min. Mass spectrometry (MS) signals were recorded at 5-second intervals to simultaneously track the evolution of oxygen (m/z = 32 and 16) and carbon dioxide (m/z = 44 and 28). The e^-/O_2 ratio was subsequently calculated by correlating the instantaneous gas evolution rates with the applied current. For the *in situ* DEMS and *in situ* XRD measurements, the P50@KO_2 GDL was preloaded with 80 μL of KO_2/G1 suspension.

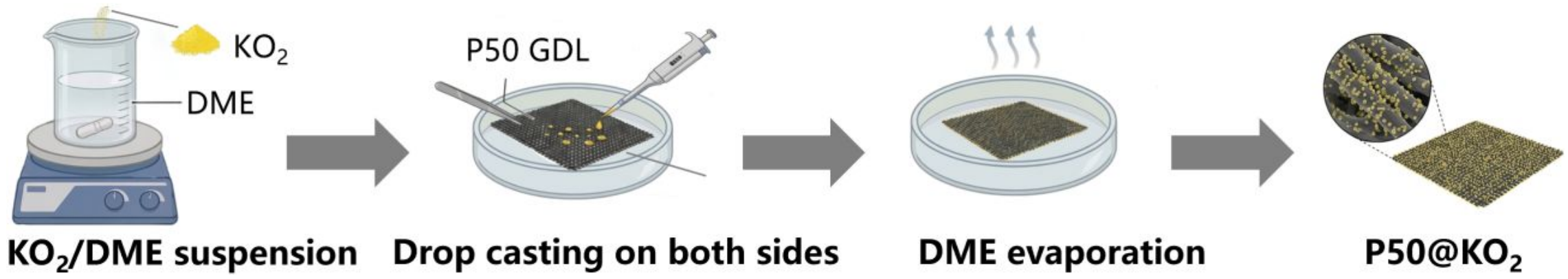

Figure S1. Schematic illustration of the KO_2-preloaded P50 GDL preparation process in an inert atmosphere.

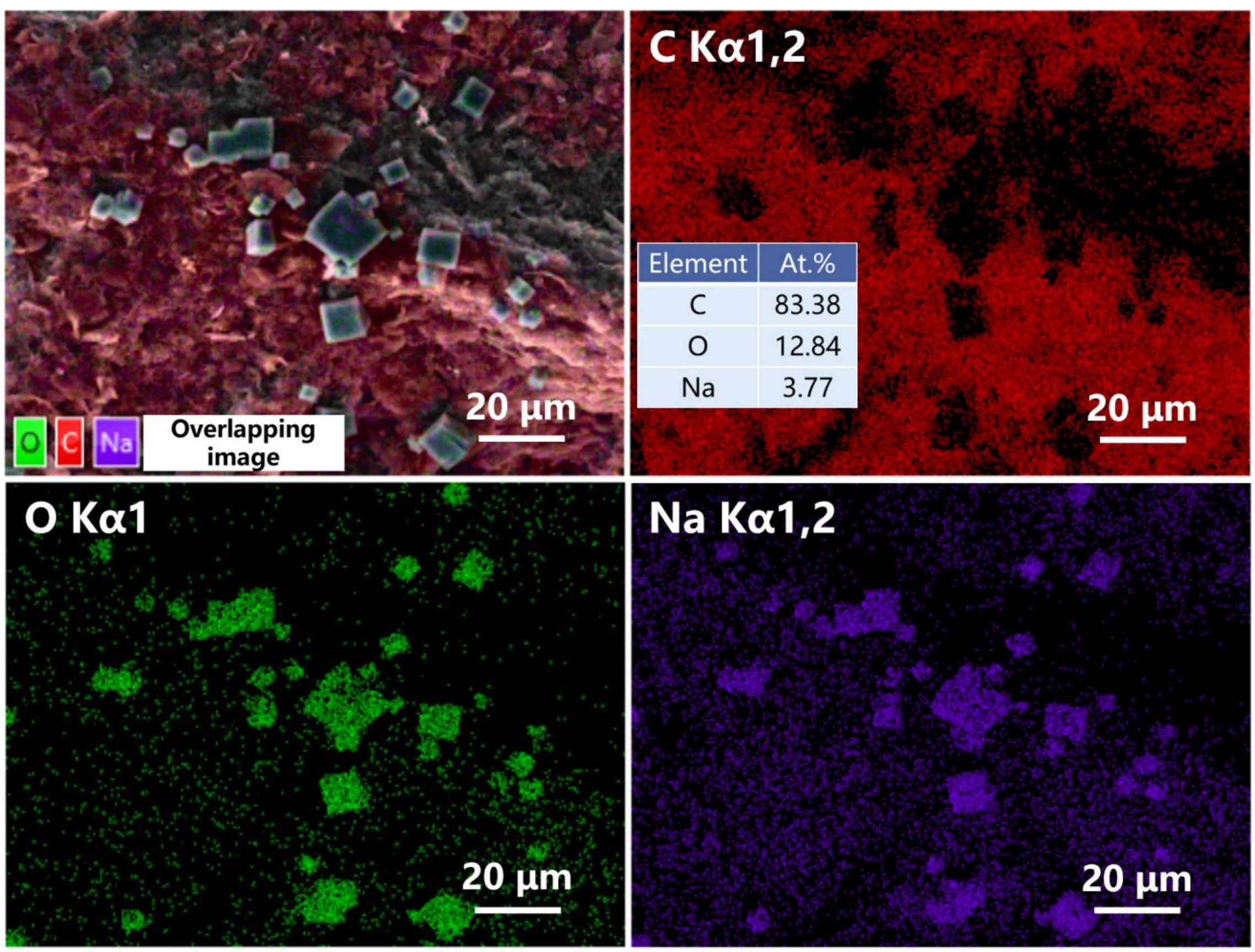

Figure S2. EDS mapping results of the discharged P50 cathode GDL at a DOD of 1 mAh. The electrolyte is 0.5 M NaOTf/G2 (05NG).

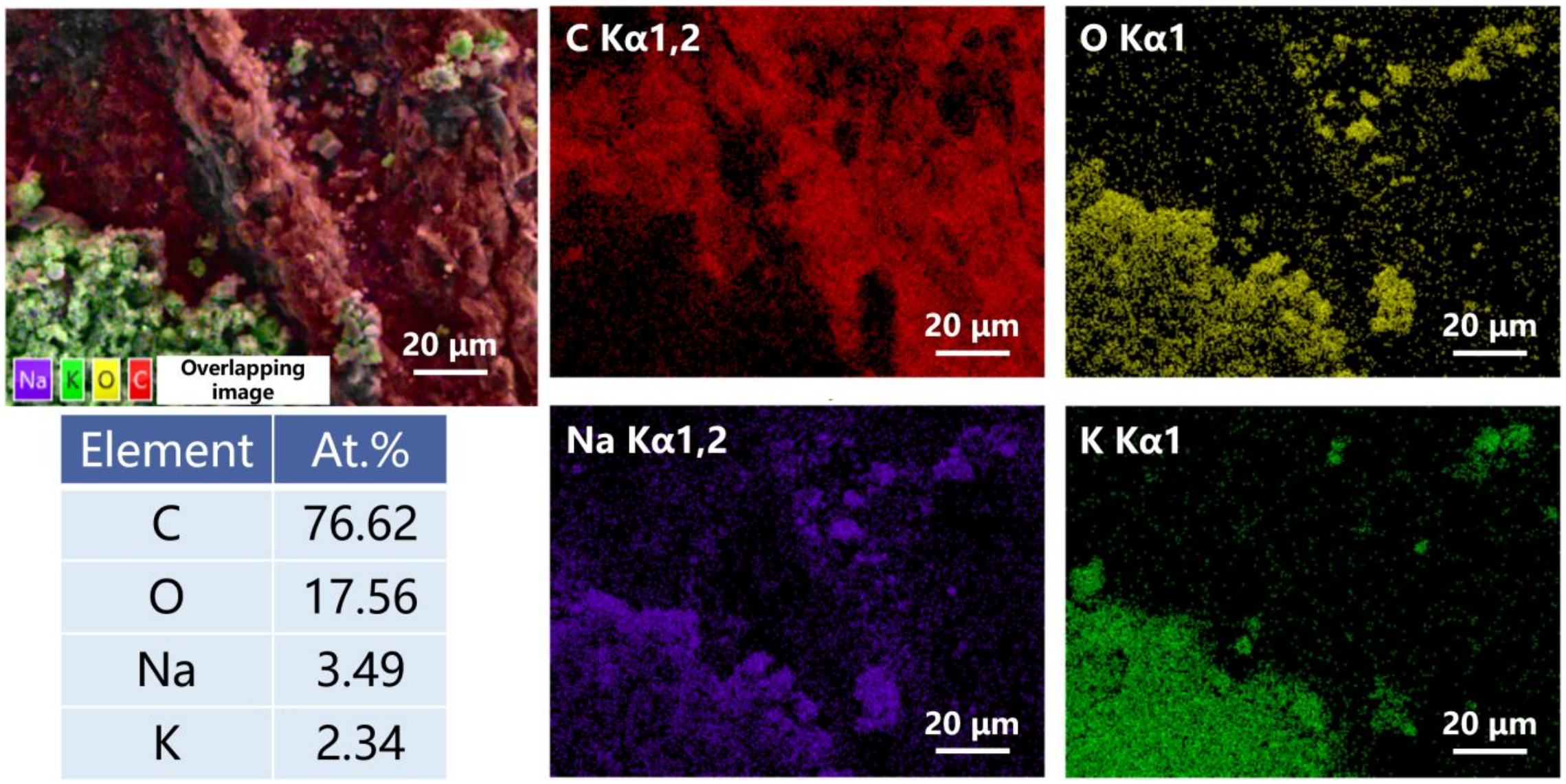

Element	At.%
C	76.62
O	17.56
Na	3.49
K	2.34

Figure S3. EDS mapping results of the discharged P50@KO_2 cathode GDL at a DOD of 1 mAh. The electrolyte is 05NG.

The spatial co-localization of K and O elemental signals confirms that the commercial KO_2 crystals exist as granular aggregates within the preloaded GDL.

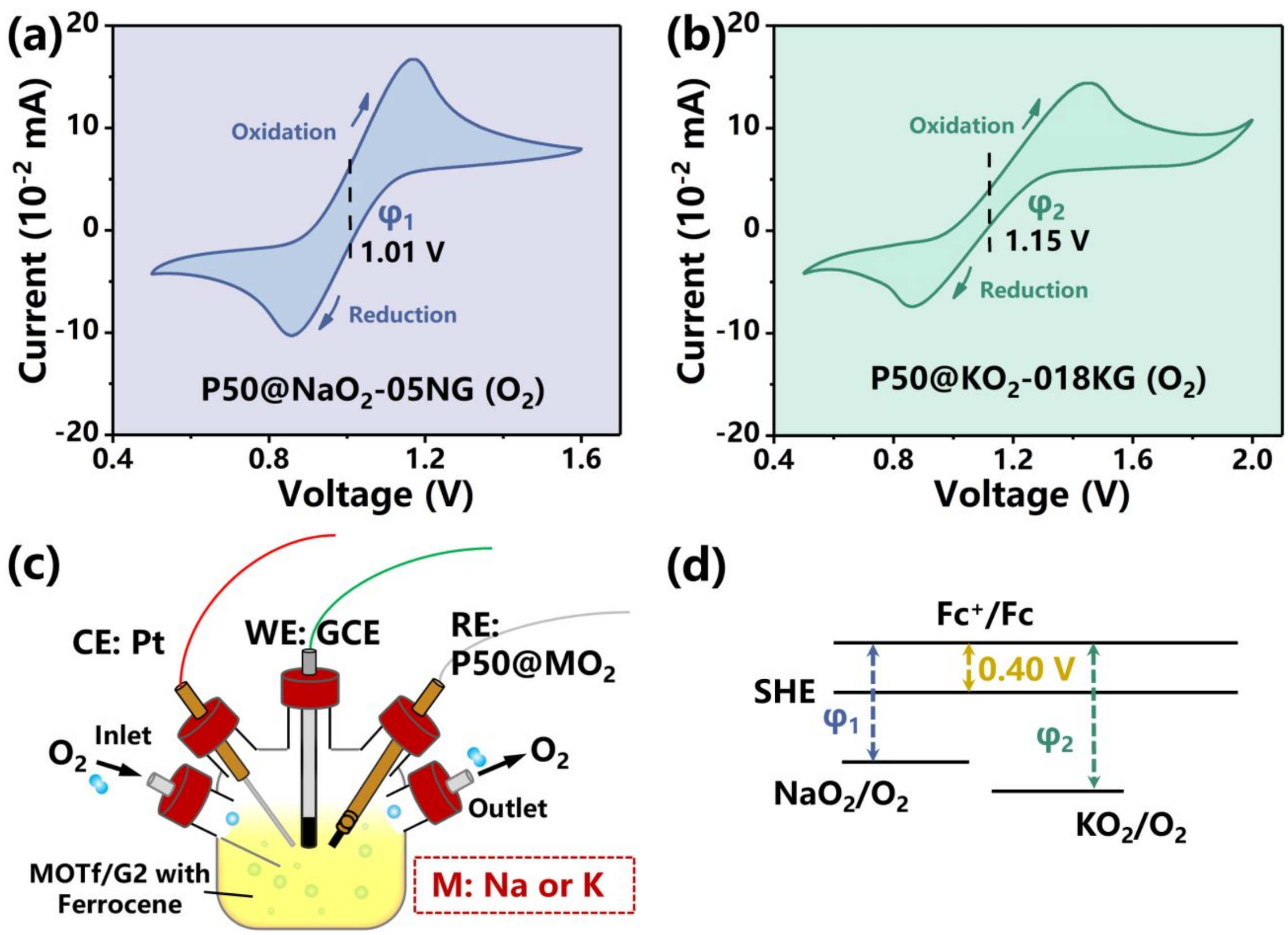

Figure S4. Electrochemical determination of superoxide/O_2 redox potentials. Potential measurements using a three-electrode configuration consisting of a glassy carbon working electrode, a platinum wire counter electrode, and (a) a P50@NaO_2 or (b) a P50@KO_2 reference electrode. Measurements were conducted in 05NG or 018KG electrolytes using the ferrocenium/ferrocene (Fc^+/Fc) as an internal standard for the Na- and K-based systems, respectively. (c) Schematic of the three-electrode cell assembly. (d) Proposed mechanism for probing the redox potentials of NaO_2/O_2 and KO_2/O_2 vs. the standard hydrogen electrode (SHE).

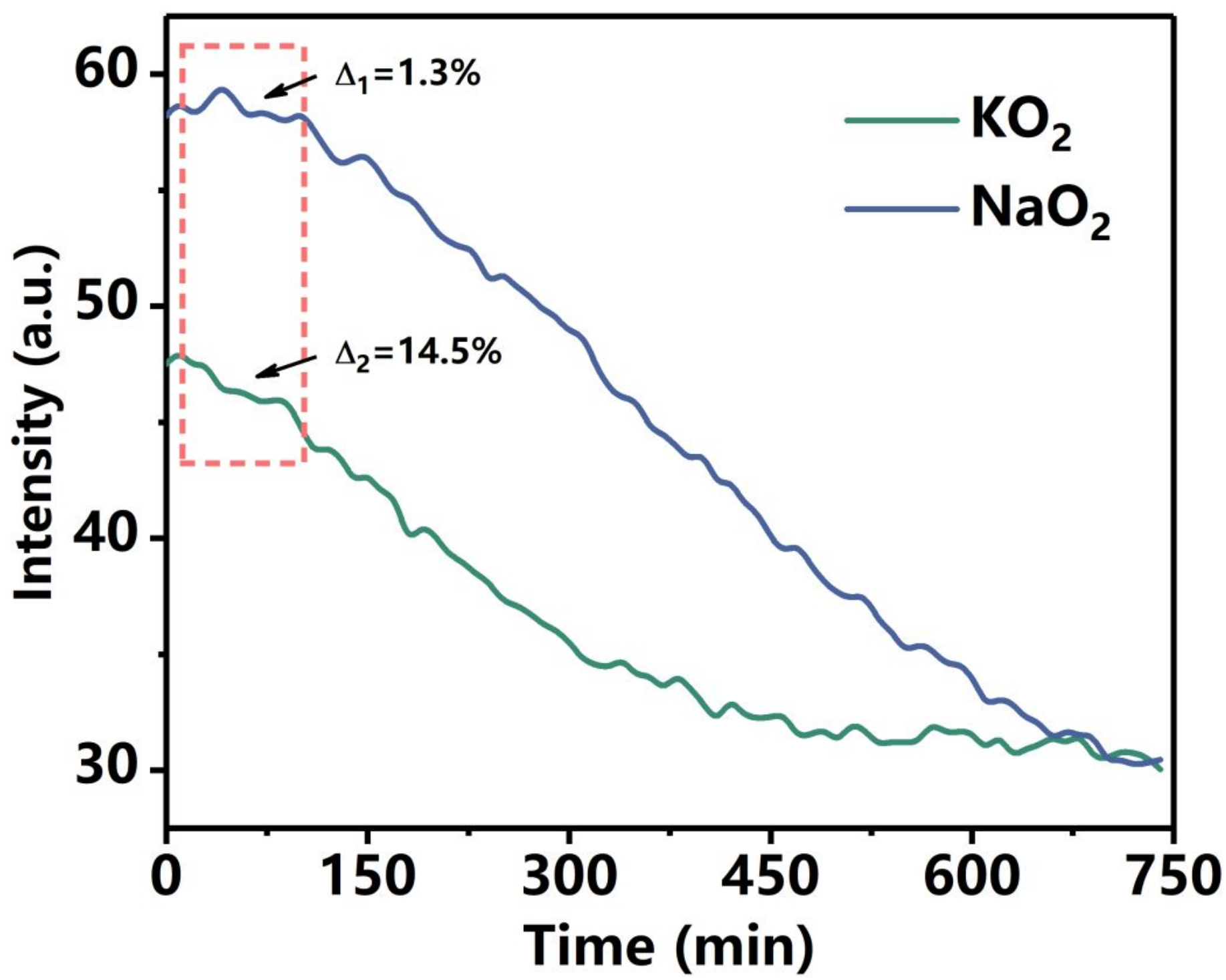

Figure S5. Intensity evolution of XRD peaks attributed to KO_2 (2θ = 31.1°) and NaO_2 (2θ = 32.3°) during the charge process.

The diffraction reflection at 31.1° is indexed to the (110) plane of KO_2, while the peak at 32.3° corresponds to the (200) plane of NaO_2.

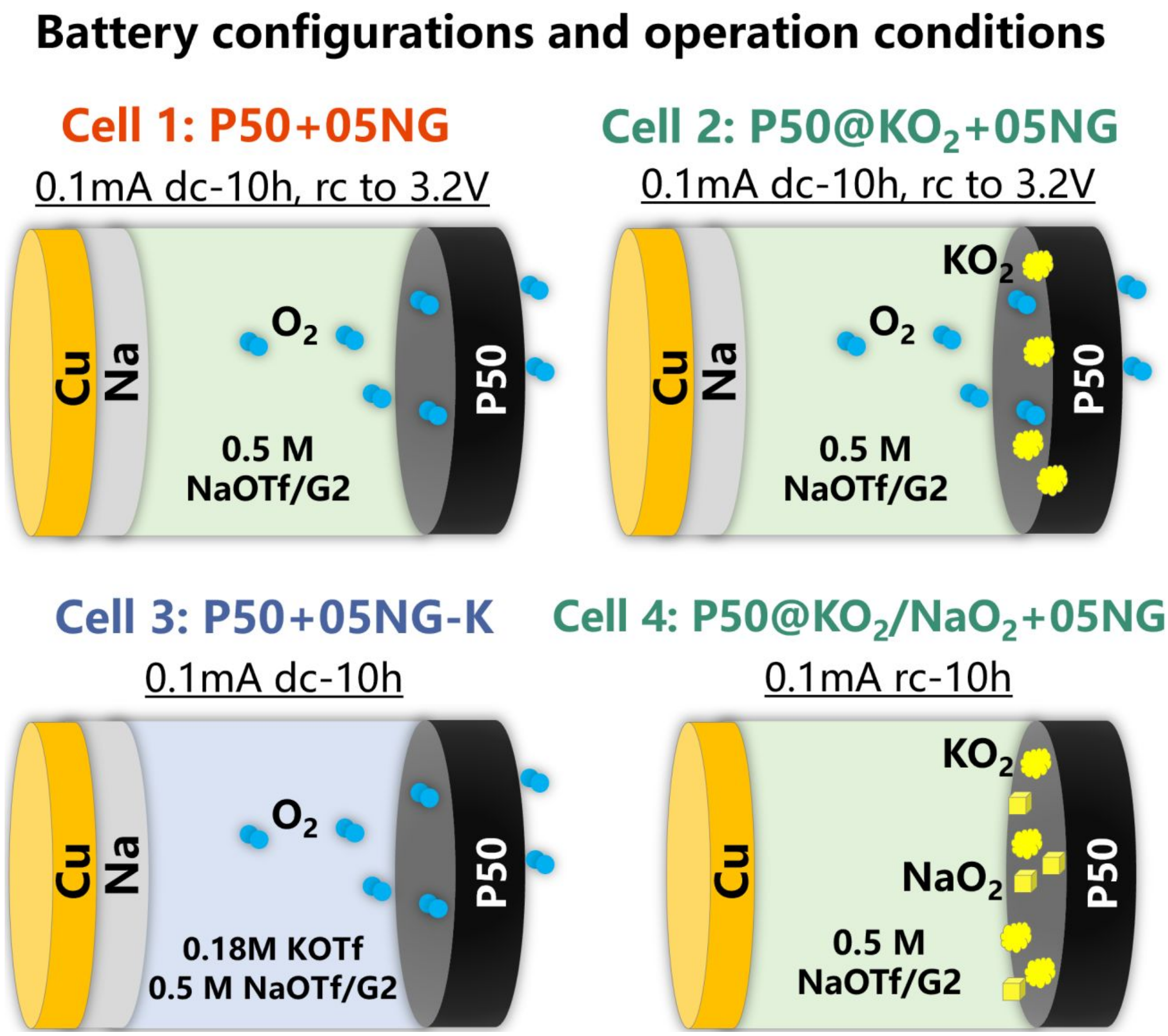

Figure S6. Schematic configurations of the assembled cells under various experimental conditions. Modular architectures for Cells 1–4 are illustrated. The configurations of Cells 1, 2, and 3 correspond to the electrochemical data presented in Figure 3a, while Cells 3 and 4 represent the experimental setups utilized for the mechanistic studies in Figure 3d.

Based on a suspension concentration of 20 mg/mL and a loading volume of 150 µL, the mass of preloaded KO_2 was determined to be approximately 3 mg. Assuming complete decomposition of the KO_2 reservoir within the electrolyte (240 µL), the theoretical K^+ concentration is roughly estimated at 0.18 mol/L.

Furthermore, the capacity contribution attributed to KO_2 oxidation is estimated at 1.11 mAh (as derived from **Figure 3**a), yielding an experimental specific capacity of 370 mAh/g. This value closely approaches the theoretical gravimetric capacity of 377 mAh/g_{KO2}, confirming the high utilization efficiency of the preloaded superoxide species.

The negligible K signal (0.02 mg/L) detected in the 05NG & P50 system is likely attributable to instrumental background noise or detection limits (**Figure 3**b).

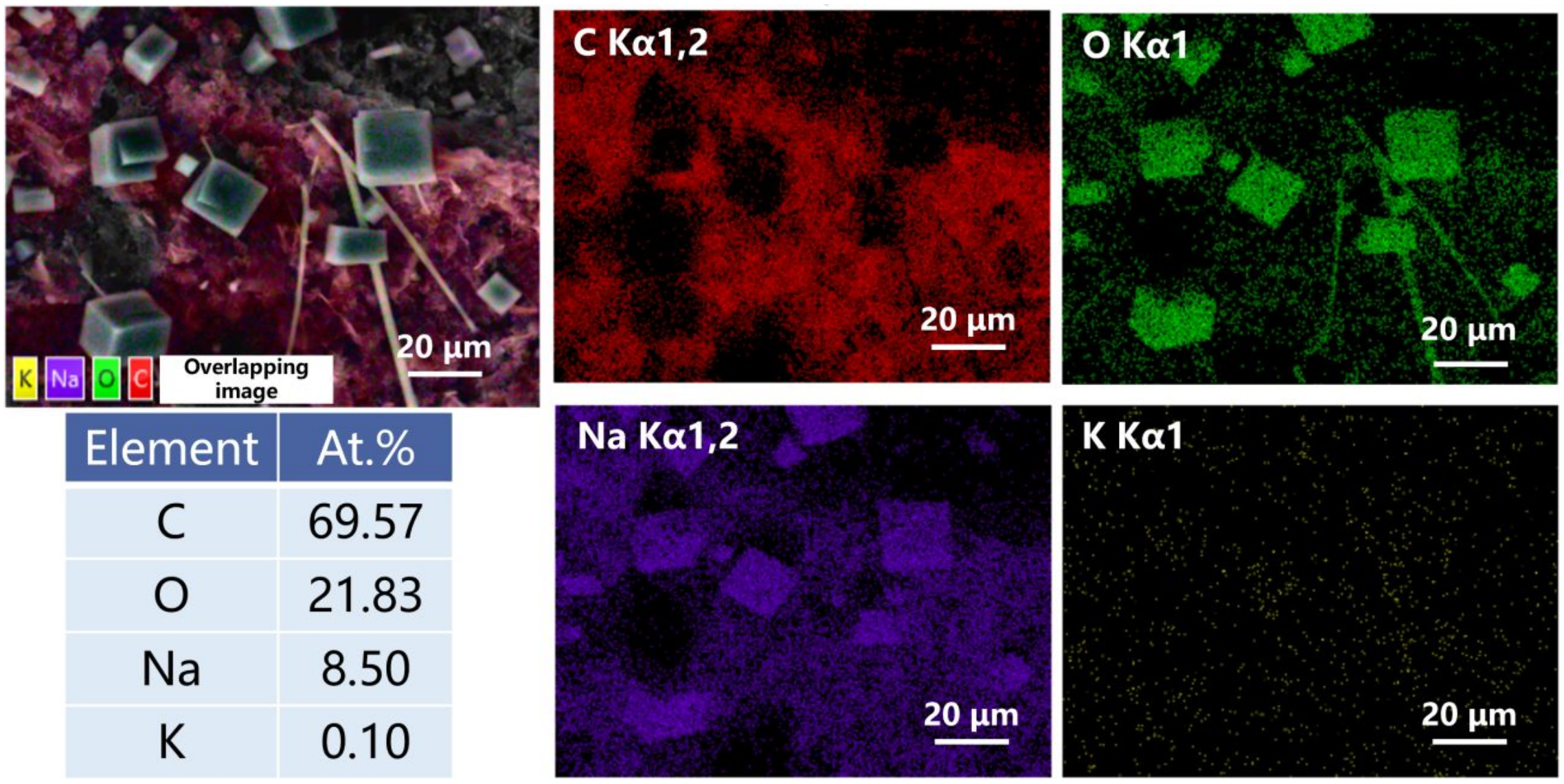

Element	At.%
C	69.57
O	21.83
Na	8.50
K	0.10

Figure S7. EDS mapping results of the discharged P50 cathode GDL at a DOD of 1.0 mAh. The electrolyte is 05NG+0.18 M KOTf (05NG-K).

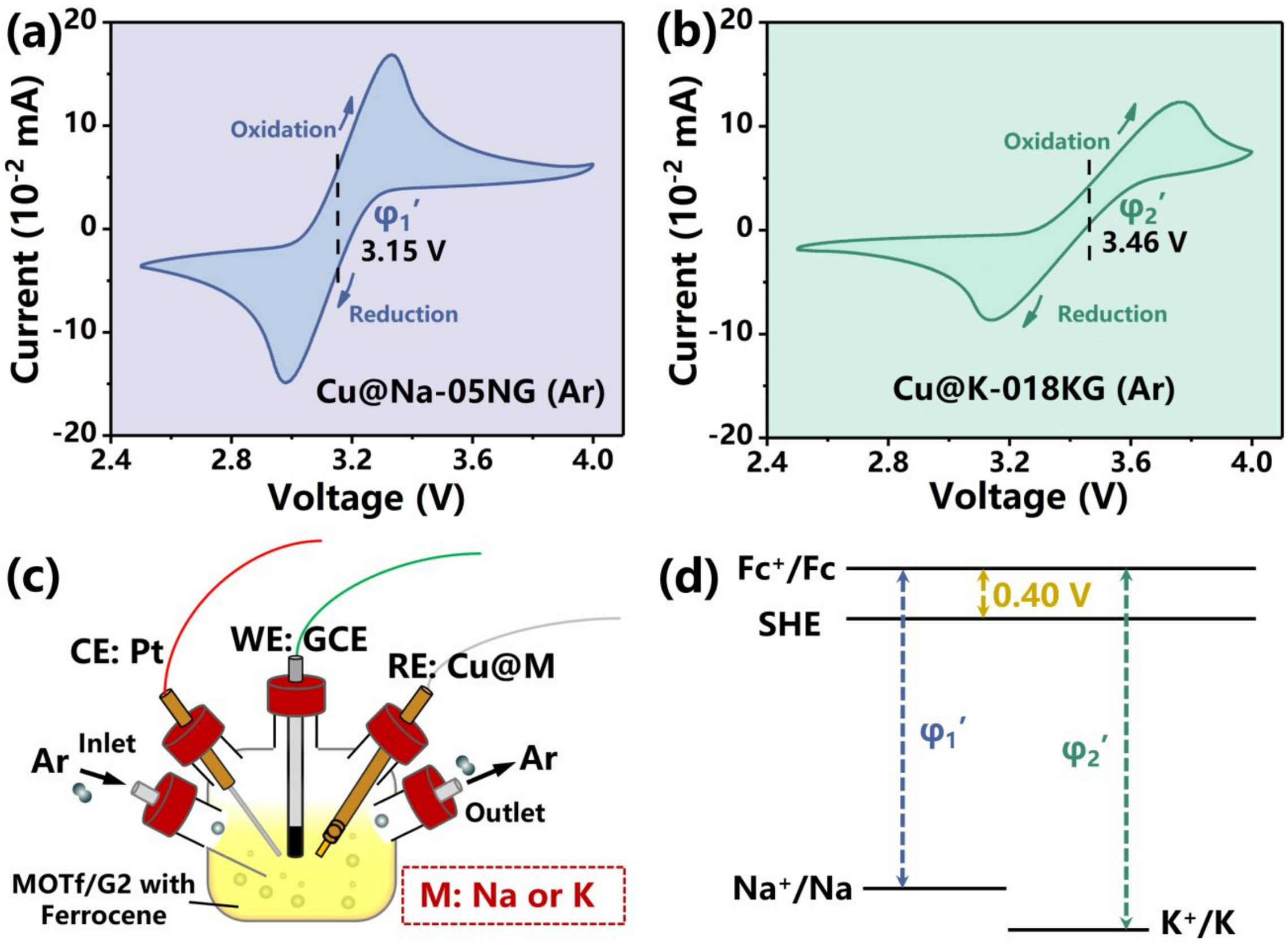

Figure S8. Electrochemical calibration of M^+/M (M = Na, K) redox potentials. Potential determination using a three-electrode configuration comprising a glassy carbon working electrode, a platinum wire counter electrode, and (a) a Cu@Na or (b) a Cu@K reference electrode. The electrolytes consist of 05NG or 018KG formulations, each containing the Fc^+/Fc as an internal standard. (c) Schematic representation of the proposed three-electrode cell assembly. (d) Proposed mechanism for probing and correlating the Na^+/Na and K^+/K redox potentials against SHE.

The equilibrium potentials of the Na^+/Na and K^+/K redox couples were first calibrated against the Fc^+/Fc internal standard using a three-electrode configuration (**Figure S8**a–c). The determined potentials are -2.75 V and -3.06 V vs. SHE, respectively (**Figure S8**d). The more noble (less negative) potential of Na^+/Na underscores a thermodynamic preference for Na^+ reduction over K^+. Consequently, despite the more reducing nature of K, the proposed Na–O_2 battery, which utilizes a K-free anode, maintains robust Na plating/stripping behavior even in the presence of trace K^+ ions.

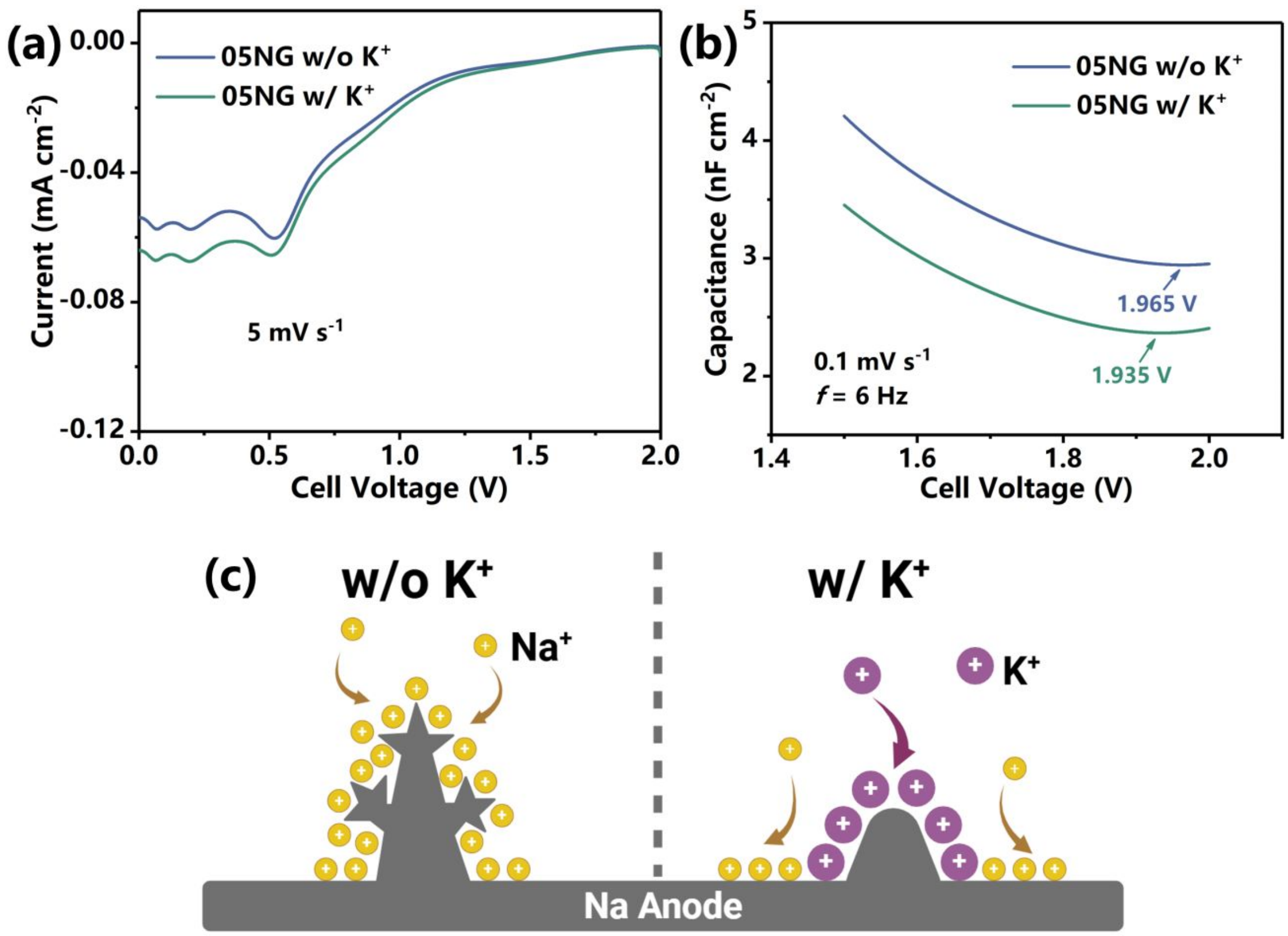

Figure S9. Influence of trace K^+ on the interfacial electrochemistry of Na anodes in Na–O_2 batteries. (a) LSV profiles of Na–Cu cells characterizing the initial interfacial processes. (b) ACV-derived differential capacitance-voltage profiles for the Cu electrode recorded within the 1.5–2.0 V window (vs. Na^+/Na). (c) Schematic depiction of the Na plating dynamics on the anode substrate. The left and right panels illustrate the deposition behavior in 05NG (without K^+) and 05NG-K (with K^+) electrolytes, respectively, highlighting the role of K^+ in modulating the nucleation morphology.

Linear sweep voltammetry (LSV) measurements on Na–Cu cells exhibit a distinct attenuation in current density below 1.5 V, signifying the onset of solid electrolyte interphase (SEI) formation on the Cu substrate (**Figure S9**a). The close resemblance of LSV profiles between 05NG and 05NG-K electrolytes suggests that the presence of K^+ has a negligible impact on the fundamental SEI buildup. To further probe the interfacial dynamics, alternating current voltammetry (ACV) was employed to derive the differential capacitance from the imaginary component of the complex impedance (**Figure S9**b). The potential of zero charge (PZC), identified at the capacitance minimum, exhibits a cathodic shift from 1.965 V to 1.935 V upon the addition of K^+, accompanied by a reduction in capacitance from 2.94 to 2.36 nF cm^{-2}. These perturbations in PZC and capacitance underscore the preferential adsorption of K^+ ions, which effectively modulates the surface charge distribution. This induces an electrostatic shielding effect that suppresses dendrite proliferation by promoting a more spatially uniform and planar Na deposition (**Figure S9**c).

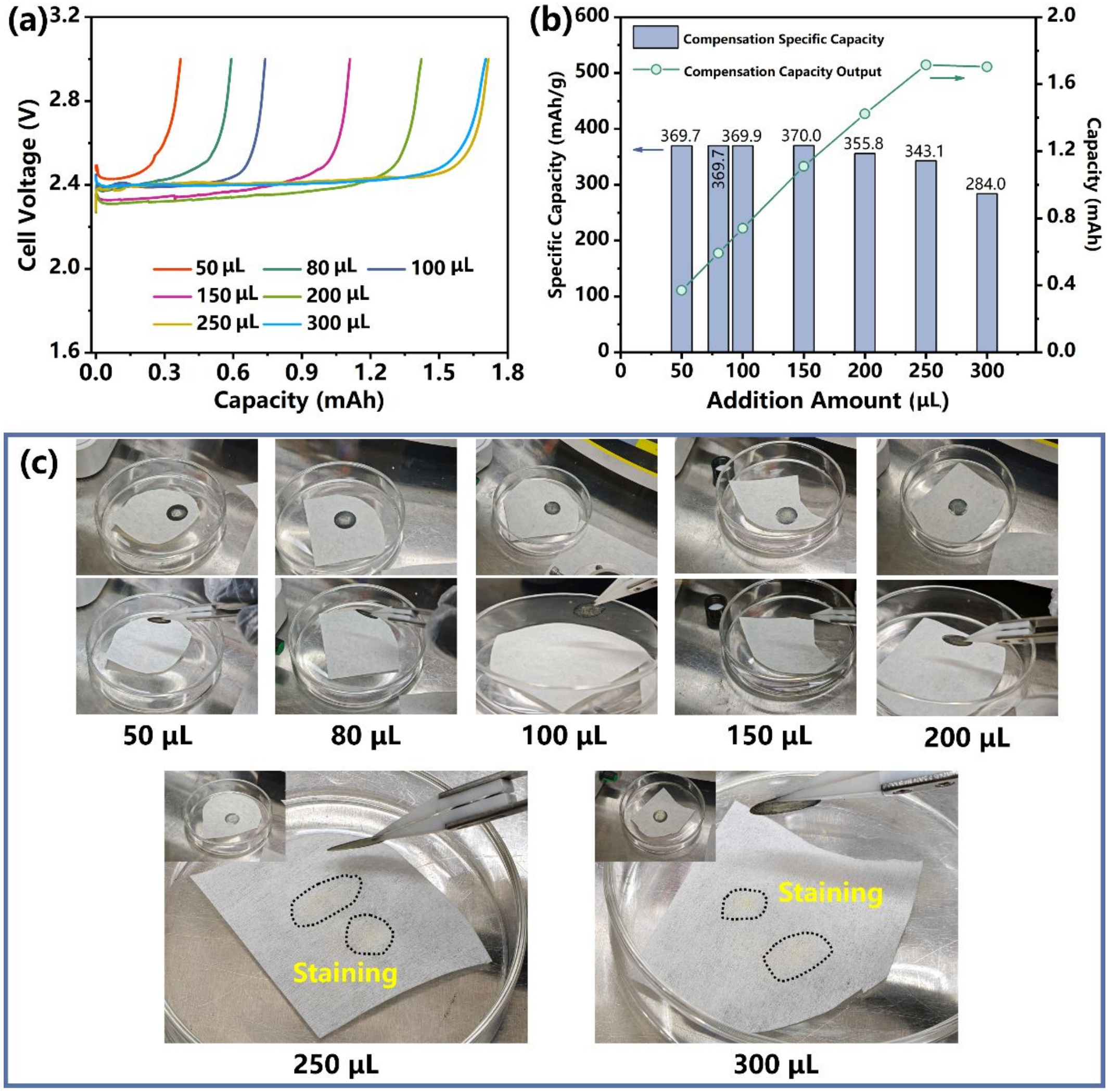

Figure S10. The relationship between KO_2/G1 suspension loading and the resulting compensation effect. (a) Typical charge voltage profiles of Na–O_2 batteries using P50@KO_2 GDLs in 05NG electrolyte. These P50@KO_2 GDLs were prepared by loading varying volumes of KO_2/G1 suspension (20 mg/ml) onto P50 substrates. (b) Charge capacity output as a function of KO_2/G1 suspension loading. The specific capacities are calculated based on the mass of KO_2 in the suspension. (c) Optical images of P50 GDLs and underlying filter papers after drop-casting varying volumes of KO_2/G1 suspension.

To optimize the compensation effect, we systematically evaluated KO_2 suspension loadings ranging from 50 μL to 300 μL. As shown in **Figure S10**, the total charge capacity scales linearly with suspension volume up to 150 μL. Beyond this threshold, the capacity output deviates from linearity, and specific capacity declines significantly. Optical examination confirms that loadings exceeding 200 μL lead to liquid leakage through the P50 gas diffusion layer, as evidenced by discernible staining on the underlying filter paper.

For long-term cycling of Na–O_2 batteries, we selected 150 μL to maximize the KO_2 reservoir's compensation efficiency. For *in situ* characterization, we chose 80 μL to roughly match the KO_2 reservoir capacity (~0.59 mAh) with the target NaO_2 capacity (~0.64 mAh).

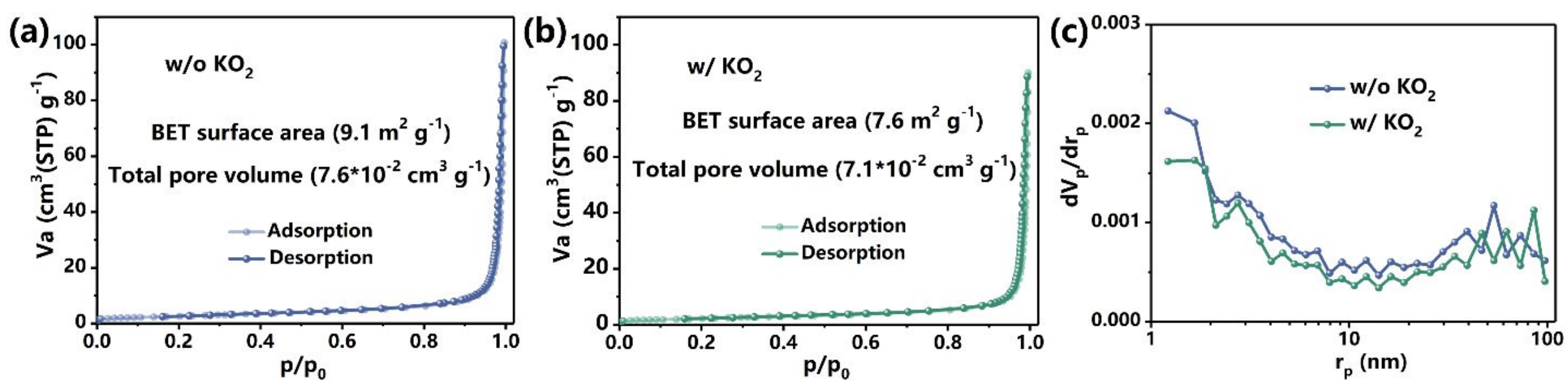

Figure S11. Microstructure analysis of GDLs. The N_2 adsorption-desorption isotherms of (a) pristine P50 GDLs and (b) KO_2-preloaded P50 GDLs. (c) Corresponding pore size distributions derived from the BJH method.

Nitrogen adsorption-desorption measurement analysis indicates that KO_2 loading causes a minor decrease in the BET surface area (from 9.1 to 7.6 m^2 g^{-1}) and pore volume (from 0.076 to 0.071 cm^3 g^{-1}). The BJH pore size distribution curves confirm that the microstructure (1–100 nm) remains largely unaffected.

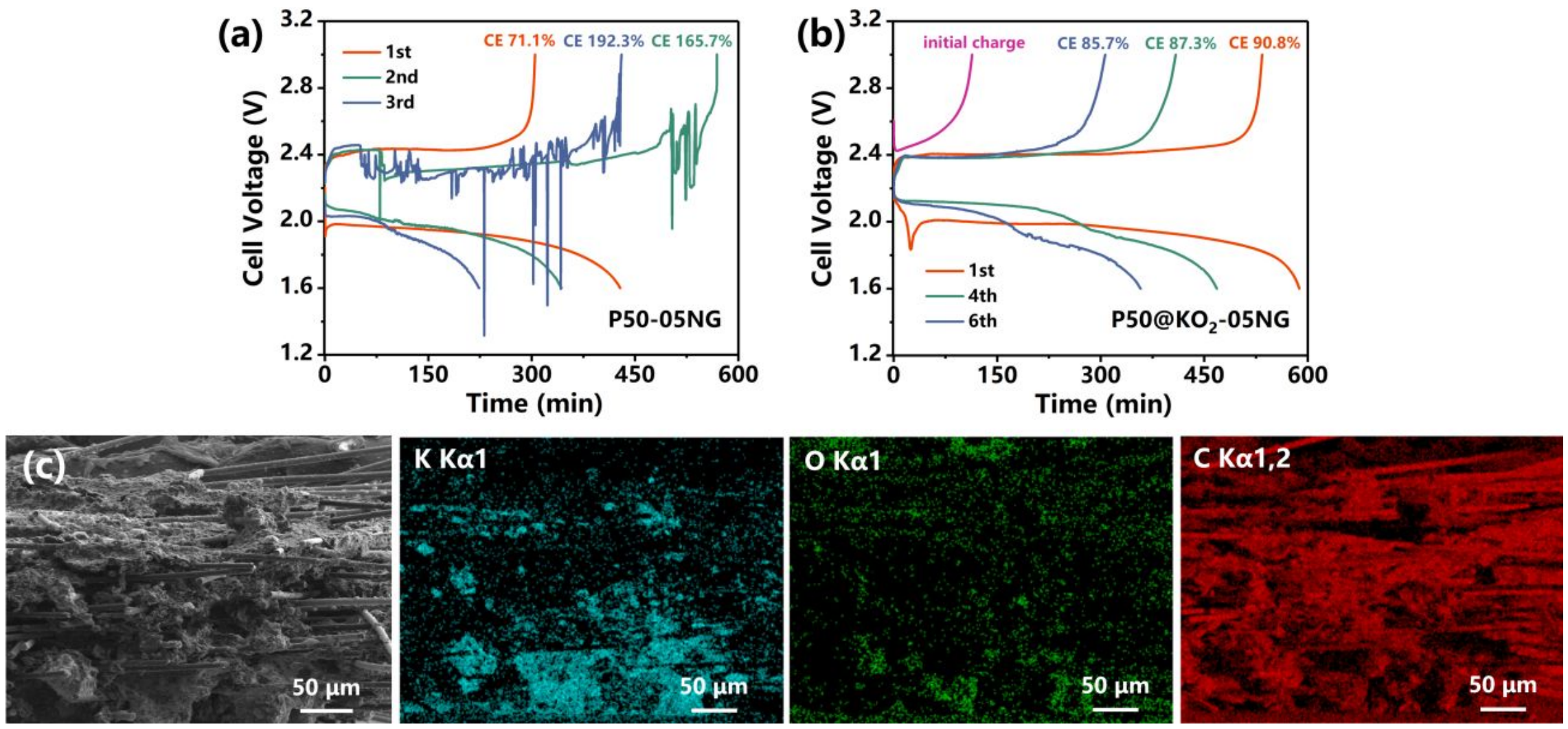

Figure S12. Comparative electrochemical evaluation and morphological analysis. Full discharge-charge performance estimation of Na–O_2 batteries with (a) P50 GDL and (b) P50@KO_2 GDL. Tests were conducted at a current density of 0.4 mA/cm^2 in 05NG electrolyte. (c) Typical cross-sectional SEM image and the corresponding EDS elemental mapping of the P50@KO_2 GDL, illustrating the distribution of the KO_2 species within the porous GDL matrix.

The Na–O_2 battery without KO_2 lasts 3 cycles with recurring micro-short circuits from the 2nd cycle onward, as evidenced by CEs larger than 100% and discernible voltage fluctuations. In comparison, the Na–O_2 battery with P50@KO_2 possesses much enhanced capacity output and capacity retention with stable voltage profiles during the cell operation.

Notably, the initial discharge process of the Na–O_2 battery employing the P50@KO_2 GDL was impeded. This phenomenon is likely attributable to the significant occupation of void spaces within the GDL by the preloaded KO_2, as evidenced by the cross-sectional SEM/EDS analyses. The resultant reduction in porosity stifles the efficient mass transfer of active species (O_2 and Na^+), leading to prohibitive polarization at a high current density of 0.4 mA/cm^2. However, the porous architecture is restored upon KO_2 decomposition during the initial charging process. This efficient recovery of void space facilitates stable cycling at deep depths of discharge of Na–O_2 batteries, significantly outperforming the pristine GDL counterpart.

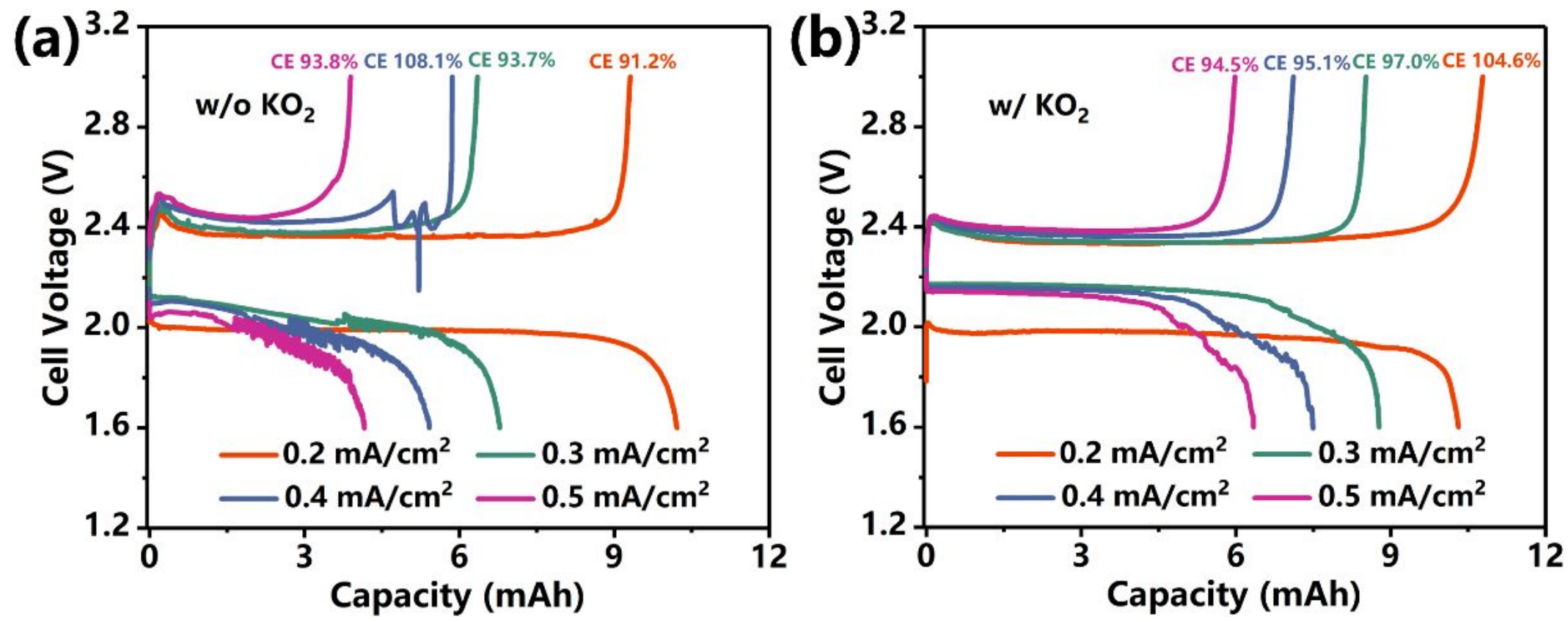

Figure S13. Rate performance and polarization analysis. Discharge/charge voltage profiles of Na–O_2 batteries based on (a) pristine P50 GDLs and (b) KO_2-preloaded P50 GDLs at various current densities.

Table S1. Summary of key electrochemical performance metrics for Na–O_2 batteries.

Condition	Test current (mA/cm^2)	Discharge capacity (mAh)	Charge capacity (mAh)	Coulombic efficiency (%)	Discharge medium voltage (V)	Charge medium voltage (V)	Polarization (V)
GDL without KO_2	0.2	10.21	9.31	91.2	1.991	2.368	0.377
	0.3	6.78	6.35	93.7	2.022	2.377	0.355
	0.4	5.42	5.86	108.1	1.995	2.422	0.427
	0.5	4.16	3.90	93.8	2.005	2.437	0.432
GDL with KO_2	0.2	10.32	10.79	104.6	1.975	2.337	0.362
	0.3	8.78	8.52	97.0	2.152	2.338	0.186
	0.4	7.49	7.12	95.1	2.141	2.363	0.222
	0.5	6.34	5.99	94.5	2.123	2.384	0.261

We compared the rate performance of P50 and P50@KO_2 electrodes at current densities from 0.2 to 0.5 mA cm^{-2}. The pristine P50 GDL exhibits voltage instability at high current densities, and its abnormal coulombic efficiency (>100%) at 0.4 mA cm^{-2} suggests internal short-circuiting. In contrast, the P50@KO_2 electrode shows enhanced capacity retention and reduced overpotentials, confirming that KO_2 immobilization does not impair the discharge performance once the cells are pre-cycled at low current densities. These results indicate that the performance gains primarily arise from the reservoir-mediated compensation effect and the stabilizing role of liberated K^+ ions.

We also observed an “activation” phenomenon characterized by lower initial discharge voltage plateaus, which likely reflects interfacial stabilization during the first cycle. Following this activation, both electrodes exhibit increased discharge capacities at 0.4 mA cm^{-2} (increasing from 3.93 to 7.49 mAh for P50@KO_2, and 2.86 to 5.42 mAh for P50). This capacity enhancement underscores the crucial role of interfacial stabilization in improving battery performance.

Table S2. Performance comparison of $Na–O_2$ batteries reported in recent literature.

No.	Methods	Optimization	CE/%	Cycle No.	Baseline	CE/%	Cycle No.	Ref.
1	Anode engineering	M-GSS@Na//CNTs (QPE)	96.07	80	Na//CNTs (QPE)	-	23	[3]
2		CP@Na//CP (SSE)	93.5	160	Na//CP (SSE)	93.8	30	[4]
3	Electrolyte optimization	Na//N-doped C (0.5 M NaOTf/G1:G4(4:1))	91.1	100	Na//N-doped C (0.5 M NaOTf/G1:G4(5:0))	54	40	[5]
4		Na//Super-P +CMC@TGP-H-060 (1 M NaOTf+0.01 M TBAOTf/G2)	95.5	60	Na//Super-P +CMC@TGP-H-060 (1M NaOTf/G2)	89.7	20	[6]
5		Na///H2315 (3.2 m NaTFSI/DMSO)	90	150	Na//H2315 (1.0 m NaTFSI/DMSO)	-	6	[7]
6	Cathode functionalization	Na//FeCoP-CC (0.5 M NaOTf/G2)	100	200	Na//CoP-CC (0.5 M NaOTf/G2)	-	60	[8]
7		Na//GNPs+10_PTFE (0.5 M NaOTf/G1)	100	142	Na//GNPs+20_PTFE (0.5 M NaOTf/G1)	100	37	[9]
**8**		**Na//P50@KO_2 (0.5 M NaOTf/G2)**	**98.5**	**600**	**Na//P50 (0.5 M NaOTf/G2)**	**88.0**	**420**	**this work**

Notes: (1) M-GSS@Na: MXene-based Na anode with sodiophilic gradient structure; QPE: PVDF–HFP@Nafion; (2) SSE: $Na_{3.25}Zr_2Si_{2.25}P_{0.75}O_{12}$; CP: Freudenberg H23; (3) sodium trifluoromethyl sulfonate (NaOTf); 1,2-dimethoxyethane (G1); tetraethylene glycol dimethyl ether (G4); (4) diethylene glycol dimethyl ether (G2); (5) TGP-H-060/H2315/P50: carbon paper; (6) CC: carbon cloth; (7) GNPs: graphene nanosheets or nanoplatelets.

The reported strategies are mainly categorized into three distinct modification avenues: anode engineering (e.g., constructing sodiophilic scaffolds), electrolyte optimization (e.g., modulating solvation structures), and cathode functionalization (e.g., integrating active electrocatalysts).

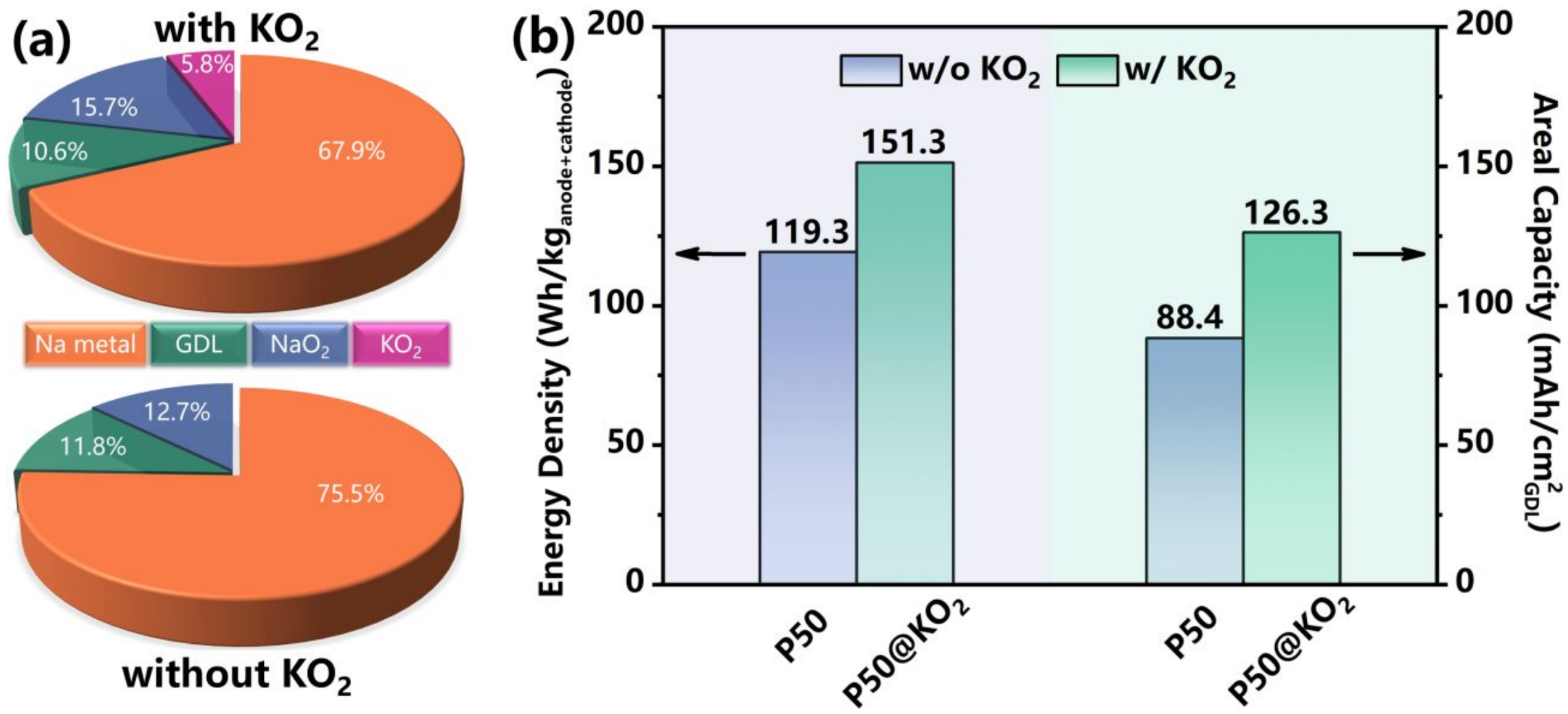

Figure S14. Capacity and energy density analysis of Na–O_2 batteries. (a) Estimated mass distribution of anode and cathode components for cells utilizing P50@KO_2 GDL (upper panel) and P50 GDL (lower panel). (b) Calculated gravimetric energy densities (left) and areal capacities (right) of Na–O_2 batteries with P50 GDLs (blue bars) and P50@KO_2 GDLs (cyan bars). Detailed parameters and calculation values are provided in Table S2.

As shown in **Figure S12**a-b, the first full discharge capacities of the Na–O_2 batteries are measured to be 3.93 mAh for the P50@KO_2 GDL and 2.86 mAh for the P50 GDL. Based on the theoretical specific capacity of NaO_2 (487.4 mAh/g), the mass of the electrochemically generated NaO_2 was calculated. The discharge products account for 15.7 wt% and 12.7 wt% of the total mass of the active electrode materials for the P50@KO_2 and P50 systems, respectively. Both values exceed the mass contribution of the GDL substrate alone.

We evaluated the energy density metrics by accounting for the total mass of all active components, including Na anode, P50 GDL, NaO_2 discharge product, and KO_2 additive (where applicable). The calculation parameters are summarized in **Table S3**. Furthermore, the cumulative areal capacity of the P50@KO_2 system is estimated to be 126.32 mAh/cm^2 over its stable lifespan (600 cycles, with a curtailed discharge capacity of 0.2 mAh and a geometric area of 0.95 cm^2). In comparison, the P50 benchmark delivers a lower cumulative capacity of 88.42 mAh/cm^2, which is primarily attributed to its limited cycle life (420 cycles).

Table S3. Parameters used for estimating the gravimetric energy densities of Na–O_2 batteries.

	Components	GDL with KO_2	GDL without KO_2
		Mass	
Na–O_2 battery system	Anode-Na (mg)	34.93	34.93
	Cathode-GDL (mg)	5.45	5.45
	Cathode-KO_2 (mg)	3.00	0.00
	Cathode-NaO_2 (mg)	8.06	5.87
	Total electrode mass (mg)	51.44	45.25
Na–O_2 battery performance	Discharge capacity (mAh)	3.93	2.86
	Discharge medium voltage (V)	1.98	1.93
	Areal capacity (mAh/cm^2)	126.32	88.42
	$E_{anode+cathode}$ (Wh/kg)	151.27	119.34

Table S4. Summary of chemical reagents and cost estimation parameters for Na–O_2 battery fabrication.

Chemicals	Information	CAS number	Price
KO_2	250g \$189.84 from Sigma-Aldrich	12030-88-5	\$7.59 × 10^{-1}/g
Metallic Na	250g \$76.65 from Aladdin	7440-23-5	\$3.07 × 10^{-1}/g
Gas diffusion layer (GDL, AvCarb® P50)	40 × 40 cm^2 \$85.00 from Fuel Cell Store	—	\$5.30 × 10^{-2}/cm^2
O_2 5N5-grade	40L \$20.13 from Air Liquide	7782-44-7	\$5.00 × 10^{-4}/mL

	Components	GDL with KO_2	GDL without KO_2
		Price	
Na–O_2 battery system	Anode-Na (\$)	1.07 × 10^{-2}	1.07 × 10^{-2}
	Cathode-GDL (\$)	5.05 × 10^{-2}	5.05 × 10^{-2}
	Cathode-KO_2 (\$)	2.27 × 10^{-3}	0
	Cathode-O_2 (\$)	5.00 × 10^{-3}	5.00 × 10^{-3}
	Total price (\$)	6.85 × 10^{-2}	6.62 × 10^{-2}
	Single discharge energy price (\$/1Wh)	8.80	11.99

Notably, we prioritize the proof-of-concept validation of the superoxide reservoir strategy and the evaluation of cathode performance in this work. Therefore, parameters such as the amount of Na metal were not specifically optimized. Besides, the GDL accounts for over 70% of the total material cost in both configurations. Our control experiments validate that the cathode GDL retains electrochemical activity even post-failure (**Figure S15**). Such a feature offers significant advantages for cost-effective cell regeneration. We anticipate that further cost reduction can be achieved through concurrent advancements in anode stabilization and electrolyte optimization, which remain subjects for future investigation.

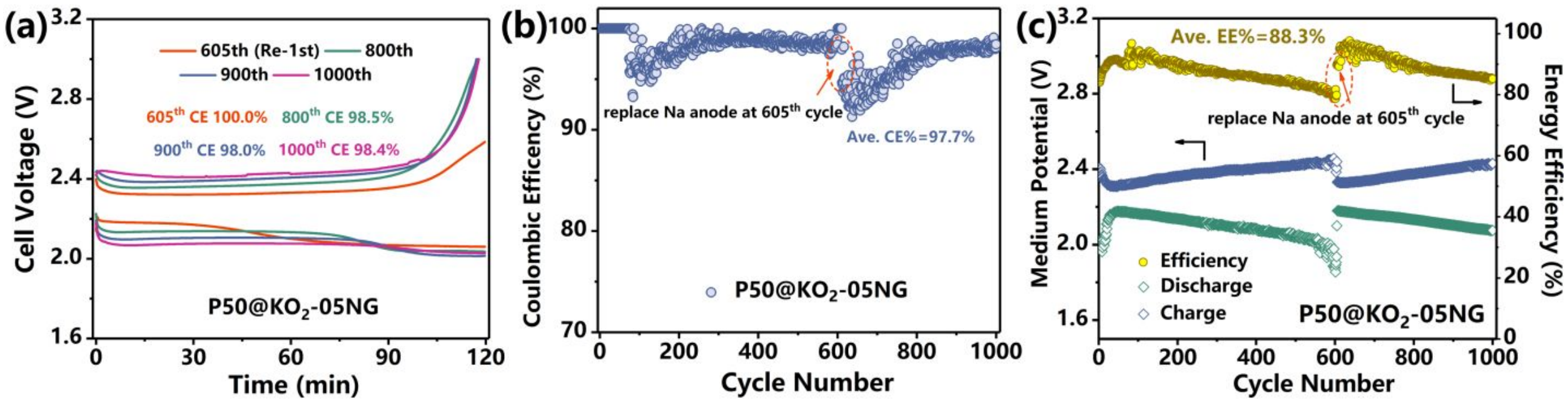

Figure S15. Evaluation of cathode cyclability via anode rejuvenation and electrolyte replenishment. (a) Representative galvanostatic voltage profiles, (b) Coulombic efficiencies, and (c) medium discharge/charge potentials with the corresponding energy efficiencies of Na–O_2 batteries utilizing a previously cycled cathode GDL. The red dashed circle highlights the electrochemical performance recovery immediately following the replacement of the Na anode and replenishment of the electrolyte, confirming the sustained activity of the cathode architecture even after the battery failure.

The benchmark Na–O_2 battery without any modification delivered 420 stable cycles, which is much better than the data reported in similar literature. We ascribe such a phenomenon to the use of a custom-engineered Swagelok-type cell with enhanced sealing integrity. This cell configuration provides a more faithful representation of intrinsic electrochemical behavior. Even against this elevated baseline, the integration of a KO_2 reservoir further extends the cycle life, underscoring its pivotal contribution to the long-term stability of the Na–O_2 battery system.

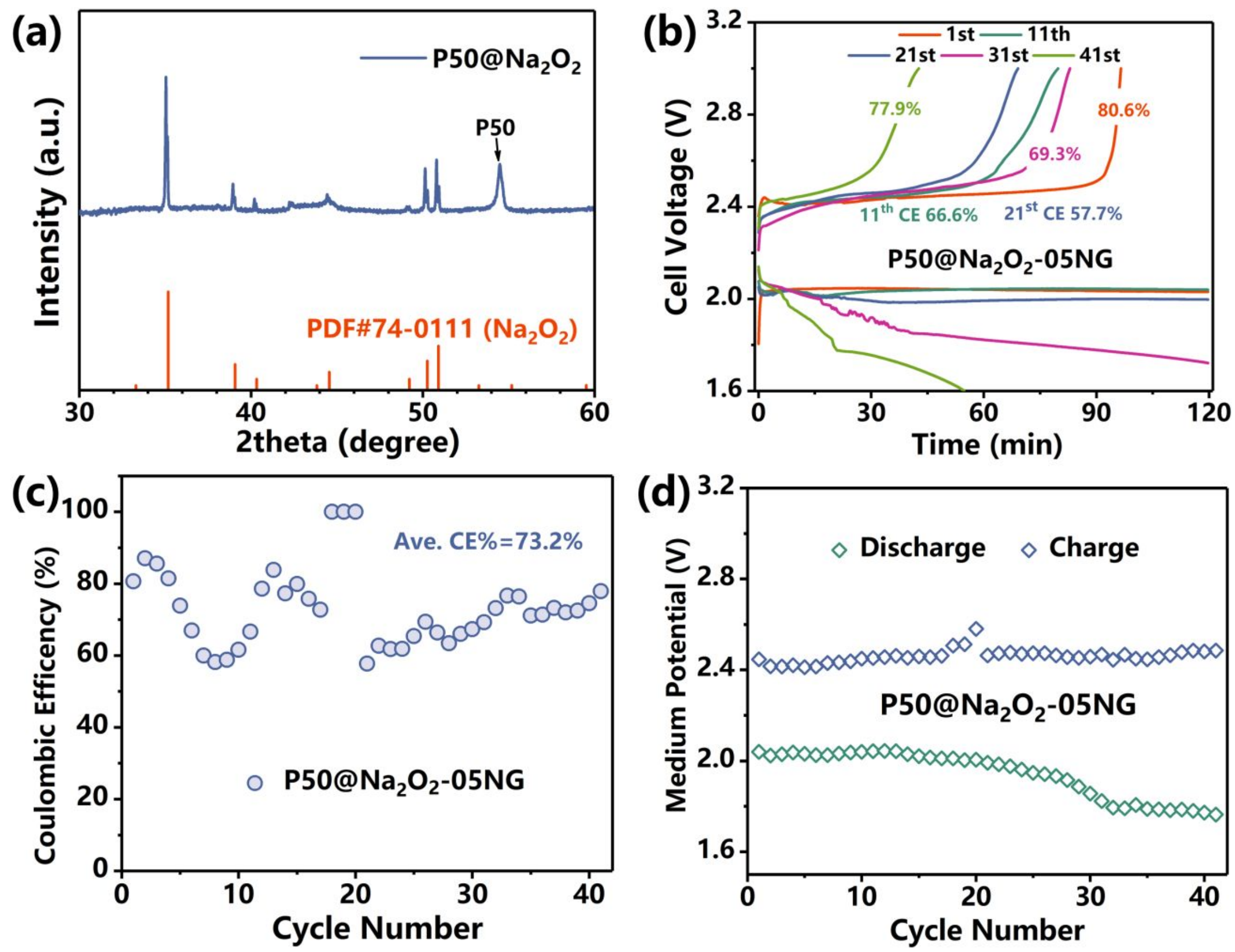

Figure S16. Structural characterization and cyclic stability of Na–O_2 batteries with Na_2O_2-preloaded cathodes. (a) XRD pattern of the P50@Na_2O_2 cathode GDL, with reflections indexed to the Na_2O_2 phase. (b) Representative galvanostatic discharge–charge profiles, (c) Coulombic efficiencies, and (d) evolution of medium discharge/charge potentials throughout the lifespan of Na–O_2 batteries using 05NG electrolyte.

Commercial Na_2O_2 was selected as the Na-containing compensation additive, serving as a surrogate due to the limited accessibility of high-purity NaO_2. The XRD pattern of the P50@Na_2O_2 GDL exhibits characteristic diffraction reflections indexed to Na_2O_2, confirming the effective integration of the additive via the drop-casting method.

Notably, the Na–O_2 battery assembled with the 05NG electrolyte and P50@Na_2O_2 GDL displays a progressive escalation in overpotentials (η) throughout its lifespan, with η values rising from 409 mV at the 10th cycle to 709 mV by the 40th cycle.

Table S5. A brief comparison between the superoxide reservoir strategy proposed for Na–O_2 batteries and the established pre-lithiation additive method for lithium-ion batteries.

Categories		Representatives	Working mechanism	Advantages	Challenges
Pre-lithiation additives for lithium-ion batteries	Li metal	Li foil, Li powder	$xLi + yM \rightarrow Li_xM_y$ where M indicates the lithium-free electrodes requiring lithiation via the chemical method	Highly efficient compensation for the active Li^+ loss and concurrently enhancing ICE and lifespan	Limited compatibility with the ambient environment and common solvents or binders; difficulty in controlling the lithiation content; requiring extra steps to remove the unreacted Li before battery assembly
	Li metal oxides	Li_5FeO_4, Li_6CoO_4, Li_2NiO_2, Li_2CuO_2	Realizing irreversible lithium release within the operation potential window of the cathode material	Enhanced chemical stability and compatibility with current battery manufacture technology	Limitations in achieving full Li^+ release; undesired gas generation; leaving transition metal oxide residues after delithiation
	Li metalloids	$Li_{22}Si_5$, $Li_{22}Ge_5$, $Li_{22}Sn_5$	Using these Li metalloids to chemically react with the target anode materials to realize pre-lithiation	High pre-lithiation capability with low redox potential and high specific capacity	High chemical reactivity; instability in ambient air; limited compatibility with polar solvents during the electrode slurry preparation and the coating process
	Li nonmetallic compounds	Li_3N, Li_2O, Li_2O_2, $Li_2C_3O_5$, $Li_2C_4O_4$, $Li_2C_4O_6$	Directly electrochemical decomposition with e^-/Li^+ release and gas evolution with the help of conducting components	High theoretical specific capacity; only gaseous evolution without any solid residue after Li^+ release	Generation of undesirable gases (e.g., CO, CO_2, N_2); limited electronic conductivities
Superoxide reservoir for Na–O_2 batteries		KO_2	Solution-mediated KO_2 decomposition with e^- release, stable spectator K^+ liberation and O_2 evolution to compensate for the irreversible capacity loss in Na–O_2 batteries	High decomposition kinetics; readily available; high specific capacity; superb compatibility with current Na–O_2 battery system	Sensitive to moisture in the ambient air; high cost from chemical vendor

Implementing these two strategies for practical applications faces significant hurdles, particularly regarding technological compatibility. There is an urgent need for materials that not only boost battery performance but also integrate seamlessly into current manufacturing practices.

References

(1) Li, Y.; Ao, H.; Xie, F.; Zhang, X.; Qin, L.; Wu, Y. Realizing Anode-Free Potassium-Organic Batteries via Sacrificial Potassium Superoxide Additives. *Energy Storage Mater.* **2026**, *86*, 105019. https://doi.org/10.1016/j.ensm.2026.105019.

(2) Lockett, V.; Sedev, R.; Ralston, J.; Horne, M.; Rodopoulos, T. Differential Capacitance of the Electrical Double Layer in Imidazolium-Based Ionic Liquids: Influence of Potential, Cation Size, and Temperature. *J. Phys. Chem. C* **2008**, *112* (19), 7486–7495. https://doi.org/10.1021/jp7100732.

(3) He, X.; Ni, Y.; Ma, W.; Zhang, Q.; Hao, Z.; Hou, Y.; Li, H.; Yan, Z.; Zhang, K.; Chen, J. PVDF–HFP@Nafion-Based Quasisolid Polymer Electrolyte for High Migration Number in Working Rechargeable Na–O_2 Batteries. *Proc. Natl. Acad. Sci. USA* **2024**, *121* (23), e2320012121. https://doi.org/10.1073/pnas.2320012121.

(4) Lin, X.; Sun, F.; Sun, Q.; Wang, S.; Luo, J.; Zhao, C.; Yang, X.; Zhao, Y.; Wang, C.; Li, R.; Sun, X. O_2 /O_2^- Crossover- and Dendrite-Free Hybrid Solid-State Na–O_2 Batteries. *Chem. Mater.* **2019**, *31* (21), 9024–9031. https://doi.org/10.1021/acs.chemmater.9b03266.

(5) Wu, C.; Yang, Q.; Zheng, Z.; Jia, L.; Fan, Q.; Gu, Q.; Li, J.; Sharma, S.; Liu, H.-K.; Yang, W.; Chen, J.; Wang, J. Boosting Na-O_2 Battery Performance by Regulating the Morphology of NaO_2. *Energy Storage Mater.* **2023**, *54*, 1–9. https://doi.org/10.1016/j.ensm.2022.10.010.

(6) Zhao, S.; Wang, C.; Du, D.; Li, L.; Chou, S.; Li, F.; Chen, J. Bifunctional Effects of Cation Additive on Na-O_2 Batteries. *Angew. Chem. Int. Ed.* **2021**, *60* (6), 3205–3211. https://doi.org/10.1002/anie.202012787.

(7) He, M.; Lau, K. C.; Ren, X.; Xiao, N.; McCulloch, W. D.; Curtiss, L. A.; Wu, Y. Concentrated Electrolyte for the Sodium–Oxygen Battery: Solvation Structure and Improved Cycle Life. *Angew. Chem. Int. Ed.* **2016**, *55* (49), 15310–15314. https://doi.org/10.1002/anie.201608607.

(8) Huang, Y.; Gan, M.; Ni, S.; Tan, C.; Gao, X.; Yu, F. Promoted Cycling Performance of Metallic Phosphide Cathode for Na-O_2 Batteries. *J. Energy Storage* **2024**, *103*, 114389. https://doi.org/10.1016/j.est.2024.114389.

(9) Enterría, M.; Letona-Elizburu, A.; Medinilla, L.; Echeverría, M.; Ortiz-Vitoriano, N. Controlling the Triple Phase Boundary on Na-O_2 Battery Cathodes with Perfluorinated Polymers. *Electrochimica Acta* **2022**, *435*, 141375. https://doi.org/10.1016/j.electacta.2022.141375.
